# E-Bayesian Estimation Using Spacing Function for Inverse Lindley Adaptive Type-I Progressively Censored Samples: Comparative Study with Applications

**DOI:** 10.1155/2024/5567457

**Published:** 2024-06-06

**Authors:** Mazen Nassar, Refah Alotaibi, Ahmed Elshahhat

**Affiliations:** ^1^ Department of Statistics Faculty of Science King Abdulaziz University, Jeddah, Saudi Arabia; ^2^ Department of Statistics Faculty of Commerce Zagazig University, Zagazig, Egypt; ^3^ Department of Mathematical Sciences College of Science Princess Nourah bint Abdulrahman University, P.O. Box 84428, Riyadh 11671, Saudi Arabia; ^4^ Faculty of Technology and Development Zagazig University, Zagazig 44519, Egypt

## Abstract

For the first time, this paper offers the Bayesian and E-Bayesian estimation methods using the spacing function (SF) instead of the classical likelihood function. The inverse Lindley distribution, including its parameter and reliability measures, is discussed in this study through the mentioned methods, along with some other classical approaches. Six-point and six-interval estimations based on an adaptive Type-I progressively censored sample are considered. The likelihood and product of spacing methods are used in classical inferential setups. The approximate confidence intervals are discussed using both classical approaches. For various parameters, the Bayesian methodology is studied by taking both likelihood and SFs as observed data sources to derive the posterior distributions. Moreover, the E-Bayesian estimation method is considered by using the same data sources in the usual Bayesian approach. The Bayes and E-Bayes credible intervals using both likelihood and SFs are also taken into consideration. Several Monte Carlo experiments are carried out to assess the performance of the acquired estimators, depending on different accuracy criteria and experimental scenarios. Finally, two data sets from the engineering and physics sectors are analyzed to demonstrate the superiority and practicality of the suggested approaches.

## 1. Introduction

In life testing and reliability studies, implementing research with complete data still takes a significant investment of time, money, and human resources. However, due to the limited timeframes for product development, life testing studies have to be completed under strict time limits. Because Type-I and Type-II censoring plans are obvious to carry out, they have been widely used in survival analysis and industry life tests, among the many other censoring plans designed to get around this problem. These censoring methods are referred to as one-stage censoring plans, in addition to hybrid Type-I and hybrid Type-II censoring plans, because they prevent the removal of living units for the experiment at any time before the end of the test. See for more details, Epstein [1] and Childs et al. [2]. This leads to the proposal of numerous multistage censoring plans in the literature, which enables the researcher to remove certain still-living units following a predefined pattern. The most popular multistage censoring plan is progressive Type-II censoring (T-IIPC). It works as follows: assume that *S*_*i*_, *i*=1, 2,…, *m* are nonnegative integers such that $\sum_{i=1}^{m}S_{i}=n-m$, where *n* is the total number of items on the test. At each *i*th failure, *Z*_*i*:*m*:*n*_, for *i*=1, 2,…, *m*, *S*_*i*_ items are at random eliminated from the remaining survival items. See Balakrishnan and Cramer [3] for more additional details about the T-IIPC. The Type-I progressive hybrid censoring (T-IPHC) plan, which Kundu and Joarder [4] investigated, combines the T-IIPC and hybrid censoring schemes. It performs similarly to the T-IIPC scheme, but the test is ended at time *T*^*∗*^=min(*Z*_*m*:*m*:*n*_, *τ*), where *τ* is a prefixed threshold. Recently, two new adaptive censoring schemes have been founded. They are called adaptive Type-II progressive censoring (AT-IIPC) and adaptive Type-I progressive censoring (AT-IPC), proposed by Ng et al. [5] and Lin and Huang [6], respectively. As in the case of the T-IPHC plan, the AT-IIPC scheme ends the test at *Z*_*m*:*m*:*n*_ if *Z*_*m*:*m*:*n*_ < *τ*. Conversely, as soon as the experimental time reaches time *τ*, no additional items will be discarded from the test. All of the remaining *S*_*m*_=*n* − *S*_1_ − ⋯−*S*_*k*_ − *m* units are removed from the experiment immediately following the *m*th failure, where *k* is the observed number of failures before time *τ*. When compared to the AT-IIPC scheme, Lin and Huang [6] demonstrated that the AT-IPC plan offers a higher estimation accuracy. The AT-IPC assures the termination of the experiment at a prefixed time *τ*. Similar to the case of the T-IPHC plan, the test ends at time *τ* if *τ* < *Z*_*m*:*m*:*n*_ and all the remaining items are removed at this point. On the other hand, the lifespan experiment will keep running to monitor failures without removing living items until *τ*, if the failure time *Z*_*m*:*m*:*n*_ is reached before *τ*. Then, at time *τ*, all the remaining items are removed, given by $S^{*}=n-k-\sum_{i=1}^{m-1}S_{i}$. Adaptive censoring plans, particularly the AT-IIPC scheme, have received a lot of attention in recent years. See the work of Elshahhat and Nassar [7], Alrumayh et al. [8], Qin and Gui [9], and Alam et al. [10], among others. Conversely, the AT-IIPC design has received little attention despite being useful for statistical inference. Some studies, including but not limited to Lin et al. [11], Nassar and Dobbah [12], Okasha et al. [13], and Alam et al. [14], among others, examined some estimation challenges from some lifetime models in the presence of AT-IIPC data. For more details about other adaptive progressive censoring plans, one can refer to Ye et al. [15], Sewailem et al. [16], Panahi and Moradi [17], and Asadi et al. [18].

In order to improve the ability of conventional models to suit different kinds of data, numerous lifetime distributions have been made available recently for modeling lifetime data. The inverse Lindley (IL) distribution, which features an upside-down bathtub-shaped hazard rate function (HRF), was proposed by Sharma et al. [19] as an inverted version of the classical Lindley distribution. Assume that *Z* > 0 is a random variable following the IL distribution, consider IL(*θ*) as its abbreviation, and *θ* be a scale parameter. Then, the probability density function (PDF), reliability function (RF), and HRF, that align with *Z*, with $\bar{\theta }=\theta /\left(1+\theta \right)$, can have the following forms:

(1) $$\begin{array}{c} g\left(z;\theta \right)=\frac{\theta \bar{\theta }e^{-\frac{\theta }{z}}\left(1+z\right)}{z^{3}},\;z>0,\theta >0, \end{array}$$

(2) $$\begin{array}{c} R\left(z;\theta \right)=1-\left(1+\frac{\bar{\theta }}{z}\right)e^{-\frac{\theta }{z}}, \end{array}$$

and

(3) $$\begin{array}{c} h\left(z;\theta \right)=\frac{{\left(\frac{\theta }{z}\right)}^{2}\left(1+z\right)}{\left[z\left(1+\theta \right)\left(e^{\frac{\theta }{z}}-1\right)-\theta \right]}. \end{array}$$

Numerous authors examined several estimation problems using different censoring strategies when the IL distribution served as the parent distribution because of how straightforward the IL distribution is. Some of its estimation issues were investigated by Basu et al. [20] using a Type-I censored sample. Using binomial removals and the T-IPHC strategy, Basu et al. [21] assessed the IL model. Basu et al. [22] examined the maximum likelihood (ML), maximum product of spacing (MPS), and Bayesian estimations for the IL distribution using hybrid censored data. Hassan et al. [23] examined the calculation of the reliability parameter for the IL distribution employing ranked set sampling. Asgharzadeh et al. [24] addressed the PDF and CDF estimation problem for the IL distribution. In their study, Alotaibi et al. [25] examined the estimation of some specific life parameters of the IL distribution via the AT-IIPC data.

Researchers considered both classical and nonclassical approaches, such as ML and Bayesian estimation methods, when estimating lifetime models for a while. In recent years, many studies have appeared to show the superiority of some other techniques rather than the mentioned methods. For example, from the classical point of view, the MPS estimation introduced by Cheng and Amin [26] can provide better estimates when compared with the ML method, especially when the sample size is small or for heavy-tailed distributions. See Ng et al. [27], Basu et al. [22], and Nassar et al. [28] for more details. Furthermore, obtaining more accurate estimates via Bayesian estimation may result from utilizing the spacing function (SF) rather than the likelihood function (LF) to determine the posterior distribution, as shown in Dey et al. [29]. In the context of Bayesian estimation, Han [30] was motivated to suggest the E-Bayesian estimation approach, which treats the hyperparameters as random variables with probabilistic models in response to the challenge of identifying their values. Many studies considered the E-Bayesian methodology; see, for example, Jaheen and Okasha [31], Okasha [32], Algarni et al. [33], Han [34], and Iqbal and Yousuf [35], among others. It is evident that all studies that took into account the E-Bayesian estimation approach used the LF as the source of observed data to derive the posterior distribution of the parameters vector. As a result, we are motivated in this work to study the E-Bayesian estimation method when the posterior distribution is obtained using the SF. It is important to mention here that it is the first time to investigate the E-Bayesian estimations using the SF. Another important motivation for this work is the flexibility of the IL distribution in analyzing real data sets. One can see the superiority of the IL distribution in modeling real data sets when compared with some other distributions later in the real data section. Furthermore, this is the first time that six-point and six-interval estimates for the parameters, including the reliability metrics, of the IL distribution have been compared using the AT-IPC, which we believe is significant for practitioners and reliability experts. The objectives of this study can be listed as given below:

1. Deriving the ML estimates (MLEs) of *θ*, RF, and HRF for the IL distribution using AT-IIPC data. The approximate confidence intervals (ACIs) are also acquired.
2. Investigating the MPS estimates (MPSEs) along with the ACIs of the different parameters.
3. Studying the Bayesian estimation for the IL distribution under the squared error (SE) loss function. The Bayes estimates are computed using both LF and SF, denoted by Bayes-LF and Bayes-SF, respectively. The Bayes credible intervals (BCIs) are also acquired using both LF and SF, denoted by BCIs-LF and BCIs-SF, respectively.
4. Exploring the E-Bayesian estimation for various parameters using both LF and SF, denoted by E-Bayes-LF and E-Bayes-SF, respectively. Also, the E-BCIs are obtained based on LF and SF, denoted by E-BCIs-LF and E-BCIs-SF, respectively. Since all theoretical solutions of *θ* developed by the proposed estimation approaches cannot be represented in closed expressions, thus we shall use two well-known packages, called “{maxLik}” (by Henningsen and Toomet [36]) and “{coda}” (by Plummer et al. [37]) in *{R}* software to obtain the required estimates.
5. Using a variety of scenarios and simulation research, compare the six-point and six-interval estimations according to a set of precise standards.
6. Demonstrating the viability of the suggested techniques by exploring two applications from the domains of engineering and physics.

This is how the remainder of the paper is organized.  Section 2 examines the MLEs and ACIs for the IL distribution that use the LF, indicated by ACIs-LF. In Section 3, the MPSEs and the ACIs employing the SF, designated as ACIs-SF, are studied.  Section 4 addresses the Bayes-LF, Bayes-SF, BCIs-LF, and BCIs-SF using the Markov chain Monte Carlo (MCMC) method. The E-Bayes-LF, E-Bayes-SF, E-BCIs-LF, and E-BCIs-SF of the various parameters are looked at in Section 5. The hype-parameters selection problem is covered in Section 6. A comprehensive Monte Carlo simulation will be run in Section 7 to examine the performance of each estimate that was taken into consideration. Two real-world data sets are presented in Section 8. A few observations will be covered in Section 9.

## 2. Likelihood Approach

In this part, the ML method is considered to get the MLEs and ACI-LF of *θ*, RF, and HRF of the IL distribution using AT-IPC data. Suppose that we have $\underset{―}{\text{z}}=\left(z_{i},i=1,\ldots ,k\right)$ as an AT-IPC sample, where *z*_*i*_=*z*_*i*:*m*:*n*_ for simplicity, with progressive censoring pattern **S**=(*S*_1_,…, *S*_*m*−1_,…, *S*_*k*_), where *S*_*m*_=…, *S*_*k*_=0. Then, the LF of the observed data, for *k* ≥ 1, takes the form as follows:

(4) $$\begin{array}{c} L\left(\theta \left|\underset{―}{\text{z}}\right.\right)=C\prod_{i=1}^{k}\left\{g\left(z_{i:m:n}\right)R^{S_{i}}\left(z_{i:m:n}\right)\right\}\;R^{S^{*}}\left(\tau \right), \end{array}$$

where *C* is the normalized constant. When the AT-IPC sample is gathered from the IL population, the LF in Equation (4) using the PDF and RF given by Equations (1) and (2), respectively, can be given by the following:

(5) $$\begin{array}{c} L\left(\theta \left|\underset{―}{x}\right.\right)={\left(\theta \bar{\theta }\right)}^{k}e^{-\theta Q}\prod_{i=1}^{k}{\left[e^{\theta x_{i}}-\left(1+\bar{\theta }x_{i}\right)\right]}^{S_{i}}{\left[e^{\theta T}-\left(1+\bar{\theta }T\right)\right]}^{S^{*}}, \end{array}$$

where *x*_*i*_=*z*_*i*_^−1^, $\underset{―}{\text{x}}=\left(x_{i},i=1,\ldots ,k\right)$, *T*=*τ*^−1^, and $Q=\sum_{i=1}^{k}\left(1+S_{i}\right)x_{i}+S^{*}T$. The log-LF is as follows:

(6) $$\begin{array}{c} \begin{array}{c} l\left(\theta \left|\underset{―}{x}\right.\right)=k\;\log\left(\theta \right)+k\;\log\left(\bar{\theta }\right)-\theta Q+\sum_{i=1}^{k}S_{i}\log\left[e^{\theta x_{i}}-\left(1+\bar{\theta }x_{i}\right)\right]+S^{*}\log\left[e^{\theta T}-\left(1+\bar{\theta }T\right)\right]. \end{array} \end{array}$$

Accordingly, the MLE of *θ*, symbolized by $\hat{\theta }$ is the solution of the next equation as follows:

(7) $$\begin{array}{c} \begin{array}{c} \frac{dl\left(\theta \left|\underset{―}{x}\right.\right)}{d\theta }=\frac{k}{\theta }+\frac{k}{\theta \left(1+\theta \right)}-Q+\sum_{i=1}^{k}S_{i}x_{i}\psi \left(x_{i};\theta \right)+S^{*}T\psi \left(T;\theta \right)=0, \end{array} \end{array}$$

where $\psi \left(x_{i};\theta \right)=\frac{\overset{´}{\bar{\theta }}-e^{\theta x_{i}}}{1+\bar{\theta }x_{i}-e^{\theta x_{i}}}$ and $\overset{´}{\bar{\theta }}=1/{\left(1+\theta \right)}^{2}$. As there is no direct formula for $\hat{\theta }$, one has to employ any numerical approach to find it. After finding $\hat{\theta }$, the MLEs of the RF and HRF at time *t*, can be produced via the plug-in property from Equations (2) and (3), respectively, as outlined below:

(8) $$\begin{array}{c} \hat{R}\left(t\right)=1-\left(1+\frac{\hat{\bar{\theta }}}{t}\right)e^{-\frac{\hat{\theta }}{t}} \end{array}$$

and

(9) $$\begin{array}{c} \hat{h}\left(t\right)=\frac{{\left(\frac{\hat{\theta }}{t}\right)}^{2}\left(1+t\right)}{\left[t\left(1+\hat{\theta }\right)\left(e^{\frac{\hat{\theta }}{t}}-1\right)-\hat{\theta }\right]}. \end{array}$$

The exact distribution of $\hat{\theta }$ is difficult to determine, which makes creating the interval estimation difficult. Therefore, we used the large sample theory to compute the required interval ranges. Via the asymptotic traits of the MLE, the ACI-LF of *θ* can be obtained as follows:

(10) $$\begin{array}{c} \hat{\theta }\pm z_{\alpha /2}\;\sqrt{{\hat{V}}_{\theta }}, \end{array}$$

where *z*_*α*/2_ is the upper (*α*/2)th percentile point of the standard normal distribution, and

(11) $$\begin{array}{c} {\hat{V}}_{\theta }={\left.{\left[-\frac{d^{2}l\left(\theta \left|\underset{―}{\text{x}}\right.\right)}{d\theta^{2}}\right]}^{-1}\right|}_{\theta =\hat{\theta }}, \end{array}$$

with

(12) $$\begin{array}{c} \frac{d^{2}l\left(\theta \left|\underset{―}{x}\right.\right)}{d\theta^{2}}=-\frac{2k}{\theta^{2}}+\frac{k}{{\left(1+\theta \right)}^{2}}-\sum_{i=1}^{k}S_{i}x_{i}\overset{´}{\psi }\left(x_{i};\theta \right)-S^{*}T\overset{´}{\psi }\left(T;\theta \right), \end{array}$$

where

(13) $$\begin{array}{c} \overset{´}{\psi }\left(x_{i};\theta \right)=\frac{\overset{´}{\overset{´}{\bar{\theta }}}-x_{i}e^{\theta x_{i}}}{1+\bar{\theta }x_{i}-e^{\theta x_{i}}}-\frac{x_{i}{\left(e^{\theta x_{i}}-\overset{´}{\bar{\theta }}\right)}^{2}}{{\left(1+\bar{\theta }x_{i}-e^{\theta x_{i}}\right)}^{2}},\;\overset{´}{\overset{´}{\bar{\theta }}}=-\frac{2}{{\left(1+\theta \right)}^{3}}. \end{array}$$

The ACIs-LF corresponding to *R*(*t*) and *h*(*t*) can be readily calculated by approximating the estimated variances of $\hat{R}\left(t\right)$ and $\hat{h}\left(t\right)$ with the delta approach, presented as follows:

(14) $$\begin{array}{c} \hat{R}\left(t\right)\pm z_{\alpha /2}\;\sqrt{{\hat{V}}_{R}},\text{ and }\hat{h}\left(t\right)\pm z_{\alpha /2}\;\sqrt{{\hat{V}}_{h}}, \end{array}$$

where ${\hat{V}}_{R}=\left[{\hat{R}}_{\theta }{\hat{V}}_{\theta }{\hat{R}}_{\theta }^{⊤}\right]$, ${\hat{V}}_{h}=\left[{\hat{h}}_{\theta }{\hat{V}}_{\theta }{\hat{h}}_{\theta }^{⊤}\right]$, and

(15) $$\begin{array}{c} {\hat{R}}_{\theta }=\frac{\hat{\bar{\theta }}e^{-\frac{\hat{\theta }}{t}}\left[\varsigma \left(1+\hat{\theta }\right)+t\right]}{t^{2}\left(1+\hat{\theta }\right)} \end{array}$$

and

(16) $$\begin{array}{c} {\hat{h}}_{\theta }=\frac{\hat{\theta }\varsigma \left\{e^{-\frac{\hat{\theta }}{t}}\left[2t+\hat{\theta }\left(\theta +\varsigma \right)\right]-\left(2t+\hat{\theta }\varsigma \right)\right\}}{t^{2}{\left[\hat{\theta }+t\left(1+\hat{\theta }\right)\left(1-e^{-\frac{\hat{\theta }}{t}}\right)\right]}^{2}}, \end{array}$$

where *ς*=1+*t*.

## 3. Product of Spacing Approach

The MPSEs and ACIs-SF of *θ*, RF, and HRF for the IL distribution are studied in this section. Many authors investigated the theoretical properties of the MPS method, including invariance and asymptotic properties, see Anatolyev and Kosenok [38] and Ghosh and Jammalamadaka [39]. Based on an observed AT-IPC sample $\underset{―}{\text{z}}=\left(z_{i},i=1,\ldots ,k\right)$, with progressive censoring pattern **S**=(*S*_1_,…, *S*_*m*−1_,…, *S*_*k*_), where *S*_*m*_=…, *S*_*k*_=0. Then, the SF of the observed data, for *k* ≥ 1, can be expressed as follows:

(17) $$\begin{array}{c} P\left(\theta \left|\underset{―}{z}\right.\right)=C\prod_{i=1}^{k+1}D_{i}\prod_{i=1}^{k}R^{S_{i}}\left(z_{i:k:n}\right)\;R^{S^{*}}\left(\tau \right), \end{array}$$

where *D*_*i*_=*F*(*z*_*i*_) − *F*(*z*_*i*−1_), *F*(.)=1 − *R*(.). For the IL distribution, we can joint the SF from Equations (1), (2), and (17) as follows:

(18) $$\begin{array}{c} P\left(\theta \left|\underset{―}{x}\right.\right)=e^{-\theta Q^{*}}\prod_{i=1}^{k+1}D\left(x_{i};\theta \right)\prod_{i=1}^{k}{\left[e^{\theta x_{i}}-\left(1+\bar{\theta }x_{i}\right)\right]}^{S_{i}}{\left[e^{\theta T}-\left(1+\bar{\theta }T\right)\right]}^{S^{*}}, \end{array}$$

where $Q^{*}=\sum_{i=1}^{k}S_{i}x_{i}+S^{*}T$ and

(19) $$\begin{array}{c} D\left(x_{i};\theta \right)=\left(1+\bar{\theta }x_{i}\right)e^{-\theta x_{i}}-\left(1+\bar{\theta }x_{i-1}\right)e^{-\theta x_{i-1}}. \end{array}$$

The natural logarithm of Equations (18) is as follows:

(20) $$\begin{array}{c} p\left(\theta \left|\underset{―}{x}\right.\right)=-\theta Q^{*}+\sum_{i=1}^{k+1}\log\left[D\left(x_{i};\theta \right)\right]+\sum_{i=1}^{k}S_{i}\log\left[e^{\theta x_{i}}-\left(1+\bar{\theta }x_{i}\right)\right]+S^{*}\log\left[e^{\theta T}-\left(1+\bar{\theta }T\right)\right]. \end{array}$$

Therefore, the MPSE of *θ*, say $\tilde{\theta }$, is the solution of the next equation as follows:

(21) $$\begin{array}{c} \frac{dp\left(\theta \left|\underset{―}{x}\right.\right)}{d\theta }=-Q^{*}+\sum_{i=1}^{k+1}\frac{\overset{´}{D}\left(x_{i};\theta \right)}{D\left(x_{i};\theta \right)}+\sum_{i=1}^{k}S_{i}x_{i}\psi \left(x_{i};\theta \right)+S^{*}T\psi \left(T;\theta \right)=0 \end{array}$$

where $\overset{´}{D}\left(x_{i};\theta \right)=\vartheta \left(x_{i};\theta \right)-\vartheta \left(x_{i-1};\theta \right)$, and $\vartheta \left(x_{i};\theta \right)=\frac{\bar{\theta }x_{i}e^{-\theta x_{i}}\left(2+\theta \left(1+x_{i}\right)+x_{i}\right)}{1+\theta }$. The MPSE $\tilde{\theta }$ is the numerical solution of Equations (21), which cannot be obtained in explicit form. Utilizing the invariance trait of the MPSE, the MPSEs of RF and HRF can be calculated, respectively, as

(22) $$\begin{array}{c} \tilde{R}\left(t\right)=1-\left(1+\frac{\tilde{\bar{\theta }}}{t}\right)e^{-\frac{\tilde{\theta }}{t}} \end{array}$$

and

(23) $$\begin{array}{c} \tilde{h}\left(t\right)=\frac{{\left(\frac{\tilde{\theta }}{t}\right)}^{2}\left(1+t\right)}{\left[t\left(1+\tilde{\theta }\right)\left(e^{\frac{\tilde{\theta }}{t}}-1\right)-\tilde{\theta }\right]}. \end{array}$$

Likewise to the ML methodology, the exact distribution of $\tilde{\theta }$ is challenging to identify, which complicates the building of the interval estimation. In this case, the ACI-SF of *θ* can be acquired by utilizing the asymptotic properties of the MPSE as follows:

(24) $$\begin{array}{c} \tilde{\theta }\pm z_{\alpha /2}\;\sqrt{{\tilde{V}}_{\theta }}, \end{array}$$

where ${\tilde{V}}_{\theta }={\left[-\frac{d^{2}p\left(\theta \left|\underset{―}{x}\right.\right)}{d\theta^{2}}\right]}^{-1}{\left|\;\right.}_{\theta =\tilde{\theta }}$, where

(25) $$\begin{array}{c} \frac{d^{2}p\left(\theta \left|\underset{―}{x}\right.\right)}{d\theta^{2}}=\sum_{i=1}^{k+1}\frac{D^{*}\left(x_{i};\theta \right)}{D^{2}\left(x_{i};\theta \right)}-\sum_{i=1}^{k+1}\frac{\overset{´}{D}\left(x_{i};\theta \right)}{D\left(x_{i};\theta \right)}-\sum_{i=1}^{k}S_{i}x_{i}\overset{´}{\psi }\left(x_{i};\theta \right)-S^{*}T\overset{´}{\psi }\left(T;\theta \right), \end{array}$$

where $D^{*}\left(x_{i};\theta \right)=D\left(x_{i};\theta \right)\overset{´}{\overset{´}{D}}\left(x_{i};\theta \right)-{\overset{´}{D}}^{2}\left(x_{i};\theta \right)$ and

(26) $$\begin{array}{c} \overset{´}{\overset{´}{D}}\left(x_{i};\theta \right)=\frac{x_{i}e^{-\theta x_{i}}\left[\theta x_{i}\left(1+x_{i}\right)\left(\theta^{2}+1\right)+\theta^{2}x_{i}\left(2x_{i}+3\right)-x_{i}-2\right]}{{\left(\theta +1\right)}^{3}}. \end{array}$$

The ACIs-SF associated with RF and HRF can be acquired as follows:

(27) $$\begin{array}{c} \tilde{R}\left(t\right)\pm z_{\alpha /2}\;\sqrt{{\tilde{V}}_{R}},\text{ and }\tilde{h}\left(t\right)\pm z_{\alpha /2}\;\sqrt{{\tilde{h}}_{R}}, \end{array}$$

where ${\tilde{V}}_{R}$ and ${\tilde{V}}_{h}$ are approximated using the delta method as mentioned in the previous section.

## 4. Bayesian Approach

This section focuses on estimating *θ* and RF from a Bayesian perspective using the AT-IPC sample, taking into account the SE loss function. In this case, we treat the parameter *θ* as a random variable that has a prior distribution to reflect the available knowledge about it. We assume that the parameter *θ* has gamma prior distribution with hyperparameters *a*, *b* > 0. We use both LF and SF as sources of observed data to derive the posterior distribution of *θ*. The posterior distributions using LF and SF can be written, respectively, as given below:

(28) $$\begin{array}{c} H_{1}\left(\theta \left|\underset{―}{x}\right.\right)=\frac{\theta^{k+a-1}{\bar{\theta }}^{k}e^{-\theta \left(Q+b\right)}}{A_{1}}\prod_{i=1}^{k}{\left[e^{\theta x_{i}}-\left(1+\bar{\theta }x_{i}\right)\right]}^{S_{i}}{\left[e^{\theta T}-\left(1+\bar{\theta }T\right)\right]}^{S^{*}} \end{array}$$

and

(29) $$\begin{array}{c} H_{2}\left(\theta \left|\underset{―}{x}\right.\right)=\frac{\theta^{a-1}e^{-\theta \left(Q^{*}+b\right)}}{A_{2}}\prod_{i=1}^{k+1}D\left(x_{i};\theta \right)\prod_{i=1}^{k}{\left[e^{\theta x_{i}}-\left(1+\bar{\theta }x_{i}\right)\right]}^{S_{i}}{\left[e^{\theta T}-\left(1+\bar{\theta }T\right)\right]}^{S^{*}}, \end{array}$$

where *A*_1_ and *A*_2_ are the normalized constants given, respectively, by the following:

(30) $$\begin{array}{c} A_{1}=\int_{0}^{\infty }\theta^{k+a-1}{\bar{\theta }}^{k}e^{-\theta \left(Q+b\right)}\prod_{i=1}^{k}{\left[e^{\theta x_{i}}-\left(1+\bar{\theta }x_{i}\right)\right]}^{S_{i}}{\left[e^{\theta T}-\left(1+\bar{\theta }T\right)\right]}^{S^{*}}d\theta \end{array}$$

and

(31) $$\begin{array}{c} A_{2}=\int_{0}^{\infty }\theta^{a-1}e^{-\theta \left(Q^{*}+b\right)}\prod_{i=1}^{k+1}D\left(x_{i};\theta \right)\prod_{i=1}^{k}{\left[e^{\theta x_{i}}-\left(1+\bar{\theta }x_{i}\right)\right]}^{S_{i}}{\left[e^{\theta T}-\left(1+\bar{\theta }T\right)\right]}^{S^{*}}d\theta . \end{array}$$

Let *ϖ*(*θ*) is any function of the unknown parameter *θ*, and we need to find its Bayes-LF and Bayes-SF using the SE loss function from the posterior distributions in Equations (28) and (29), denoted by ${\hat{\varpi }}_{BL}\left(\theta \right)$ and ${\hat{\varpi }}_{BS}\left(\theta \right)$, respectively. In this situation, the Bayes estimators are obtained by finding the expectation of the posterior distributions, respectively, as follows:

(32) $$\begin{array}{c} {\hat{\varpi }}_{BL}\left(\theta \right)=\int_{0}^{\infty }\varpi \left(\theta \right)H_{1}\left(\theta \left|\underset{―}{x}\right.\right)d\theta \end{array}$$

and

(33) $$\begin{array}{c} {\hat{\varpi }}_{BS}\left(\theta \right)=\int_{0}^{\infty }\varpi \left(\theta \right)H_{2}\left(\theta \left|\underset{―}{x}\right.\right)d\theta . \end{array}$$

Because the integrals in Equations (32) and (33) are quite difficult, the Bayes estimators cannot be obtained explicitly. For computing the Bayes estimates as well as the BCIs, we implement the MCMC approach with the Metropolis–Hastings (M–H) process. The computation operations are described in Algorithm 1 to obtain the Bayes-LF.

Using Algorithm 1, but by replacing the MPSE as a starting value instead of MLE and employing the posterior distribution in Equation (29), one can obtain the Bayes-SF and BCIs-SF of *θ*, *R*(*t*), and *h*(*t*), presented by ${\hat{\theta }}_{BS}$, ${\hat{h}}_{BS}\left(t\right)$, and ${\hat{h}}_{BS}\left(t\right)$, respectively, of the IL distribution using AT-IPC data.

## 5. E-Bayesian Approach

In standard Bayesian estimation, the values of the hyperparameters are defined either arbitrarily by the investigator or based on experience. These values are treated as constants. Conversely, these hyperparameters are viewed by the E-Bayesian method of estimation as random variables with determined probability distributions. As an outcome, the main benefit of E-Bayesian estimation is that it employs the expectation of the regular Bayes estimators to take into consideration all potential values of the hyperparameters. Let *ϖ*(*θ*) be an unknown parameter, and its Bayes estimator is determined as $\hat{\varpi }\left(\theta \right)$. Also, assume that *h*(*a*, *b*) is the joint prior distribution for the hyperparameters *a* and *b*. Then, as indicated by Han [30], the E-Bayes estimator of *ϖ*(*θ*) can therefore be obtained as shown below:

(34) $$\begin{array}{c} \tilde{\varpi }\left(\theta \right)=\int \int_{\Omega }\hat{\varpi }\left(\theta \right)h\left(a,b\right)\;da\;da, \end{array}$$

where *Ω* is the domain of *a* and *b*. As pointed out by Han [30], the hyperparameter prior distributions have to be established to make sure that the prior distribution of the unknown parameter *θ* is a decreasing function in *θ*. One can easily see that when 0 < *a* < 1 and *b* > 0, the gamma distribution can accomplish this attribute. As a result, we select the prior distribution of the hyperparameter *a* to be the beta distribution. On the other hand, the prior distribution of the hyperparameter *b* is selected to be a uniform distribution on the interval (0, *c*). Then, the joint prior distribution of the hyperparameters can be obtained as follows:

(35) $$\begin{array}{c} h\left(a,b\right)=\frac{a^{{ɛ}_{1}-1}{\left(1-a\right)}^{{ɛ}_{2}-1}}{cB\left({ɛ}_{1},{ɛ}_{2}\right)},0<a<1,0<b<c,{ɛ}_{1},{ɛ}_{2}>0. \end{array}$$

Using the aforementioned assumptions, the E-Bayes estimators of *ϖ*(*θ*) using both Bayes-LF and Bayes-SF in Equations (32) and (33), which use the LF and SF approaches, respectively, can be expressed as follows:

(36) $$\begin{array}{c} {\tilde{\varpi }}_{BL}\left(\theta \right)=\int_{0}^{c}\int_{0}^{1}{\hat{\varpi }}_{BL}\left(\theta \right)\;h\left(a,b\right)da\;db \end{array}$$

and

(37) $$\begin{array}{c} {\tilde{\varpi }}_{BS}\left(\theta \right)=\int_{0}^{c}\int_{0}^{1}{\hat{\varpi }}_{BS}\left(\theta \right)h\left(a,b\right)\;da\;db. \end{array}$$

Due to the complex nature of the original Bayes estimators, it is not as simple to obtain the E-Bayes estimators, as expected. As a result, we generate samples from the joint prior distribution of the hyperparameters in Equation (35) and then use them to get samples from the target posterior distributions using the M–H algorithm. The steps listed in Algorithm 2 show how to collect samples and subsequently get the required E-Bayes-LF.

The same steps in Algorithm 2 can be used to get the E-Bayes-SF and E-BCIs-SF of the unknown parameters using the SF. In this case, the MPSE is used as starting values and the M–H algorithm is acquired to get samples from the posterior distribution in Equation (29).

## 6. Hyperparameter Selection

Figuring out the appropriate hyperparameter value is the main difficulty in Bayesian analysis, particularly in the context of an informative prior for the density parameter. Additionally, the values of hyperparameters are chosen for the unknown parameters based on two types of information: the average expected value and the uncertainty of the unknown parameter in the model we are thinking about. Here are the steps we suggest for figuring out the values of hyperparameters *a* and *b* using previous samples, such as

Step 1: Put the true value of *θ*.
Step 2: Simulate a random sample of size *n* from IL(*θ*).
Step 3: Calculate the MLE $\hat{\theta }$ of *θ*.
Step 4: Redo Steps 2 and 3 *𝒢* times to acquire ${\hat{\theta }}^{i},\;i=1,2,\ldots ,𝒢$.
Step 5: Assign the gamma density prior's mean and variance to the sample mean and sample variance of ${\hat{\theta }}^{i}$, respectively, as
  (38) $$\begin{array}{c} E\left({\hat{\theta }}^{j}\right)\equiv E\left(\pi \left(\theta \right)\right)\;\to \;\frac{1}{B}\sum_{i=1}^{G}{\hat{\theta }}^{i}=\frac{a}{b} \end{array}$$
  and
  (39) $$\begin{array}{c} \text{Var}\left({\hat{\theta }}^{j}\right)\equiv \text{Var}\left(\pi \left(\theta \right)\right)\;\to \;\frac{1}{G-1}{\sum_{i=1}^{G}\left({\hat{\theta }}^{i}|-|G^{-1}|\sum_{i=1}^{G}|{\hat{\theta }}^{i}\right)}^{2}=\frac{a}{b^{2}}, \end{array}$$
  where *𝒢* is the number of generated samples from the IL distribution.
Step 6: The estimated hyperparameters $\overset{˘}{a}$ and $\overset{˘}{b}$ of *a* and *b* can be found directly by solving Equations (38) and (39) simultaneously, as
  (40) $$\begin{array}{c} \overset{˘}{a}=\frac{{\left(\frac{1}{G}\sum_{i=1}^{G}{\hat{\theta }}^{i}\right)}^{2}}{\frac{1}{G-1}\sum_{i=1}^{G}{\left({\hat{\theta }}^{i}|-|G^{-1}|\sum_{i=1}^{G}|{\hat{\theta }}^{i}\right)}^{2}}\text{ and }\overset{˘}{b}=\frac{\frac{1}{G}\sum_{i=1}^{G}{\hat{\theta }}^{i}}{\frac{1}{G-1}\sum_{i=1}^{G}{\left({\hat{\theta }}^{i}|-|G^{-1}|\sum_{i=1}^{G}|{\hat{\theta }}^{i}\right)}^{2}}, \end{array}$$
  respectively.
Step 7: Redo Steps 3–6 to get the values of *a* and *b* by the MPSE $\tilde{\theta }$ of *θ*.

## 7. Numerical Evaluations

To examine the actual behavior of the acquired estimators of *θ*, *R*(*t*), and *h*(*t*) derived via ML and MPS approaches as well as their extensions in Bayesian and E-Bayesian inferential approaches, extensive Monte Carlo simulations are conducted based on large 1,000 AT-IPC samples drawn from the IL (0.5) distribution. With time *t*=0.1, the actual values of *R*(*t*) and *h*(*t*) are utilized as (0.9708,1.2724). Taking *τ*(=0.5, 1.5) and *n*(=40, 80), various scenarios of *k* (effective censored-sample size) and *S*_*i*_,  *i*=1, 2,…, *k*, (progressive design) are reported in Table 1. In this table, the censoring **S** : (0, 0, 0, 1, 1, 1) (for instance) is symbolized by (0^3^, 1^3^) for simplicity. For specification, each value of *k* is considered as a failure percentage of each *n* as $\frac{k}{n}\times 100\%=50$ and 75%.

In a Bayesian setup, picking the hyperparameter values is the main problem. For this purpose, we will choose values for the hyperparameters *a* and *b* through the method of past sample data described in Section 6. In this case, we create 2,000 past-complete samples (with *n*=50) from IL (0.5). So, the values of (*a*, *b*) are taken as (93.15613, 184.0805) by the LF method as well as (93.47667, 189.0999) by the SF method. Additionally, to see how the affect of the hyperparameters *c* and *ɛ*_*i*_,  *i*=1, 2, on the E-Bayes' calculations, we generate numbers for parameters *ɛ*_*i*_,  *i*=1, 2, from the beta distribution. As a result, for fixed *c*=0.5, the values of (*ɛ*_1_, *ɛ*_2_) are taken as (99.30387, 196.191) by the LF method as well as (99.67437, 201.6223) by the SF method.

**Figure 1:**
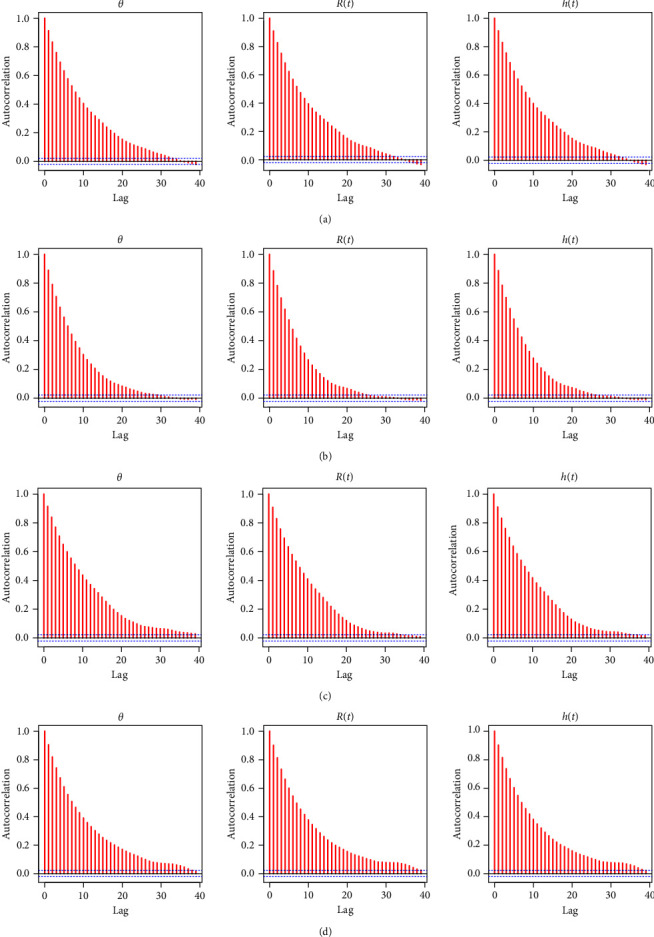
The ACF diagnostics of *θ*, *R*(*t*), and *h*(*t*) in Monte Carlo simulation: (a) Bayes-LF; (b) Bayes-SF; (c) E-Bayes-LF; (d) E-Bayes-SF.

**Figure 2:**
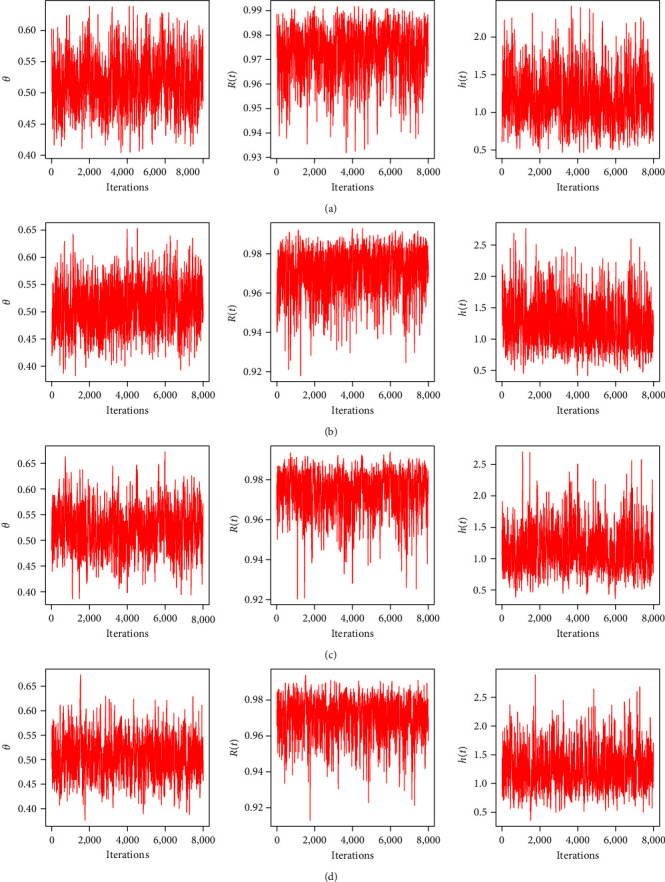
Trace diagnostics of *θ*, *R*(*t*), and *h*(*t*) in Monte Carlo simulation: (a) Bayes-LF; (b) Bayes-SF; (c) E-Bayes-LF; (d) E-Bayes-SF.

**Figure 3:**
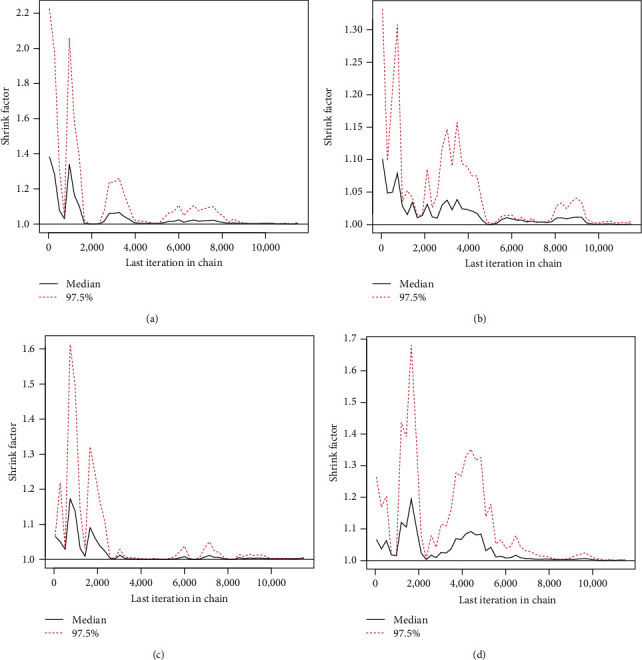
The BGR diagnostics in Monte Carlo simulation: (a) Bayes-LF; (b) Bayes-SF; (c) E-Bayes-LF; (d) E-Bayes-SF.

In order to apply the M–H method, the first 2,000 (out of 10,000) MCMC iterations of each unknown quantity are burned in. The resulting 95% BCIs and Bayes estimates for *θ*, *R*(*t*), or *h*(*t*) using the LF (or SF) approach are then computed. To find a good representative iteration from the objective posterior distributions from LF and SF approaches, based on Test (1) when *n*=40, three convergence tools are used: (1) auto-correlation function (ACF), (2) trace, and (3) Brooks–Gelman–Rubin (BGR) diagnostic; see Figures 1, 2, and 3. As a consequence, for plots based on LF or SF, Figure 1 means that the relationship between data within each group and the distribution of the results is strong and reliable; Figure 2 shows that the simulated sequences of *θ*, *R*(*t*), or *h*(*t*) are well mixed, and Figure 3 shows that the variance within the Markovian chains is about the same as the variance between them. Additionally, using the same sample created by Test (1) when *n*=40 (as an example), we shall monitor the acceptance rate of the M–H algorithm developed by the normal distribution as a proposal in all proposed estimation approaches. As a result, the acceptance rates of Bayes-LF, Bayes-SF, E-Bayes-LF, and E-Bayes-SF are 93.48%, 94.37%, 94.43%, and 93.72%, respectively. Thus, we can determine that the collected MCMC iterations give an acceptable approximation of the posterior density, and thus, the inferences derived are effective and reliable.

Specifically, the average estimate (Av.E) of *θ* (for instance) is given by the following:

(41) $$\begin{array}{c} Av.E\left(\theta \right)=\frac{1}{1,000}\sum_{j=1}^{1,000}{\overset{˘}{\theta }}^{\left[j\right]}, \end{array}$$

where ${\overset{˘}{\theta }}^{\left[j\right]}$ is the estimate of *θ* at *j*th sample. The provided estimates of *θ* are compared using their mean absolute biases (MABs), root mean squared errors (RMSEs), and average confidence lengths (ACLs) as

(42) $$\begin{array}{c} \text{RMSE}\left(\overset{˘}{\theta }\right)=\sqrt{\frac{1}{1,000}\sum_{j=1}^{1,000}{\left({\overset{˘}{\theta }}^{\left[j\right]}-\theta \right)}^{2}}, \end{array}$$

(43) $$\begin{array}{c} \text{MAB}\left(\overset{˘}{\theta }\right)=\frac{1}{1,000}\sum_{j=1}^{1,000}\left|{\overset{˘}{\theta }}^{\left[j\right]}-\theta \right|, \end{array}$$

and

(44) $$\begin{array}{c} {\text{ACL}}_{\left(1-\alpha \right)\%}\left(\theta \right)=\frac{1}{1,000}\sum_{j=1}^{1,000}\left(U_{{\overset{˘}{\theta }}^{\left[j\right]}}|-|L_{{\overset{˘}{\theta }}^{\left[j\right]}}\right), \end{array}$$

respectively, where (*ℒ*(·), *𝒰*(·)) refers to the (lower, upper) bounds of (1 − *α*)% ACI (or BCI) estimate of *θ*.

Tables 2, 3, 4, 5, 6, 7, 8, 9, and 10 show the results of the simulation. We report the following remarks based on the lowest RMSE, MAB, and ACL values, based on Tables 2, 3, 4, 5, 6, 7, 8, 9, and 10:

(1) The suggested estimating approaches have produced estimates of *θ*, *R*(*t*), or *h*(*t*) that have all behaved well. Here is our general remark.
(2) As *n* (or *k*) increases, all evaluations become even better. A similar comment is also noted when $\sum_{i=1}^{k}S_{i}$ narrowed down.
(3) As *τ* increases, the simulated RMSE, MAB, and ACL values of all estimates of *θ*, *R*(*t*), or *h*(*t*) decreased.
(4) Comparing the proposed estimation procedures, it is noted that
  - All results derived from the Bayes (or E-Bayes) methodology, due to the additional gamma information, performed superior compared to the frequentist estimates.
  - The MLE results of *θ* performed superior compared to the MPSE results, whereas the MPSE results of *R*(*t*) and *h*(*t*) performed superior compared to the MLE results.
  - The Bayes-LF results of *θ* performed superior compared to the Bayes-SF results, whereas the Bayes-SF results of *R*(*t*) and *h*(*t*) performed superior compared to the Bayes-LF results. The same observation is also reached when comparing the Bayes and E-Bayesian inferential approaches.
  - The ACI-LF results of *θ* performed superior compared to the ACI-SF results, whereas the ACI-SF results of *R*(*t*) and *h*(*t*) performed superior compared to the ACI-LF results.
  - The Bayes-LF results of *θ* performed superior compared to the Bayes-SF results, whereas the Bayes-SF results of *R*(*t*) and *h*(*t*) performed superior compared to the Bayes-LF results. The same observation is also reached when comparing the BCI and E-BCI inferential approaches.
(5) Comparing the proposed schemes reported in Table 1, it is noted that all estimates of *θ*, *R*(*t*), or *h*(*t*) provide better results based on right-censoring utilized in Tests (3) and (6) for each *n* than others.
(6) As a summary, using the investigated censored data, the E-Bayes' methodology via SF-based is recommended to explore the reliability features of the IL model.

## 8. Real-Life Applications

In this section, we deliver two actual sets of data from the domains of engineering and physics in order to illustrate how flexible and adaptable the suggested approaches are to real-world occurrences.

### 8.1. Engineering Data Analysis

**Figure 4:**
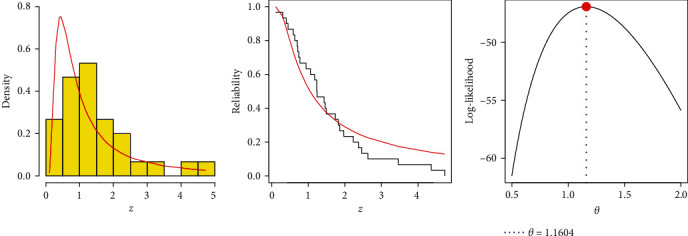
Graphics for goodness-of-fit of IL model using RME data.

Actual data collection was examined by this application, which first came to light by Murthy et al. [40].  Table 11 displays the times between failures for 30 repairable mechanical equipment (RME) components in this data collection. First, we must determine whether or not the suggested IL(*θ*) model fits the entire set of RME data. Therefore, we first calculate the Kolmogorov–Smirnov (K–S) distance and its *p*-value using the MLE. Consequently, we find that *θ* has an MLE (Std.Er) of 1.1604 (0.1619) and a K–S (*p*-value) of 0.1412 (0.5881), where Std.Er refers to the standard error. Consequently, we may conclude that the IL distribution is a good model to suitably fit the RME data. We also examine three goodness-of-fit visualizations in Figure 4: (i) plotting RME's histograms with fitted IL density line, (ii) fitted/empirical reliability lines, and (iii) log-likelihood. It validates the same fitting outcome.

Briefly, To demonstrate the usefulness and superiority of the proposed IL model, we compare its fit with the other six distributions in the literature as competitors, namely:

1. Inverted Weibull (IW (*γ*, *θ*)) proposed by Keller et al. [41],
2. Inverted Lomax (ILomax (*γ*, *θ*)) proposed by Kleiber and Kotz [42],
3. Inverted Chen (IChen (*γ*, *θ*)) proposed by Srivastava and Srivastava [43],
4. Inverted exponentiated Rayleigh (IER (*γ*, *θ*)) proposed by Ghitany et al. [44],
5. Inverted Nadarajah–Haghighi (INH (*γ*, *θ*)) proposed by Tahir et al. [45],
6. Alpha power inverted exponential (APIE (*γ*, *θ*)) proposed by Ceren et al. [46].

Besides the K–S (*p*-value), this comparison is made based on several metrics of model selection, namely: Akaike (*A*), (2) Bayesian (*B*), consistent Akaike (*CA*), Hannan–Quinn (*HQ*); see Table 12. These criteria will be evaluated through the MLEs $\hat{\gamma }$ and $\hat{\theta }$. The results reported in Table 12 show that the IL distribution has the lowest fitted values of *A*, *B*, *CA*, and *HQ*, except for the highest CP values. This fact shows that the IL lifetime model is generally better compared to other models.

Three AT-IPC samples (with *m*=15) are constructed from the whole RME data listed in Table 11, based on various options of *τ* and **S**; see Table 13.  Table 14 lists the point estimates (along their Std.Ers) and 95% interval estimates (along their widths) of *θ*, *R*(*t*), and *h*(*t*) (at *t*=0.5) for each sample in Table 13 using the LF, SF, Bayes-LF, Bayes-SF, E-Bayes-LF, and E-Bayes-SF approaches. The noninformative prior, or *a*=*b*=0,  *i*=1, 2, is used as the prior knowledge regarding the IL parameter is unavailable. In addition, we set *c*=1 and *ɛ*_*i*_=0.75 for *i*=1, 2 in order to construct the E-Bayes inferences (using both LF and SF methods). In order to assess the Bayes and E-Bayes estimates derived from the LF and SF methods presented in Sections 4 and 5, we eliminate the first 5,000 iterations of 30,000 MCMC samples for each unknown quantity, assuming that the start value of *θ* represents its frequentist value. Because we did not know the true parameter value and used the frequentist estimate as the initial value, we used a large number of iterations to get a stable chain and discarded the first 5,000 iterations as the burn-in period to remove the impact of the initial values and to guarantee the convergence of the chains. Burn-in is meant to give the Markovian chain time to achieve its posterior distribution, especially if it started with a bad guess point. To “burn-in” a chain, we simply discard the first samples with an appropriate size before making inferences; see Gelman et al. [47]. According to the results shown in Table 14, the point and interval estimates of *θ*, *R*(*t*), or *h*(*t*) that were produced using the LF/SF approaches are extremely similar to those that were created using the Bayes/E-Bayes approaches. Additionally, it is noted that the 95% ACI estimation limits that were produced using the BCI/E-BCI techniques and the LF/SF procedures are relatively similar.

**Figure 5:**
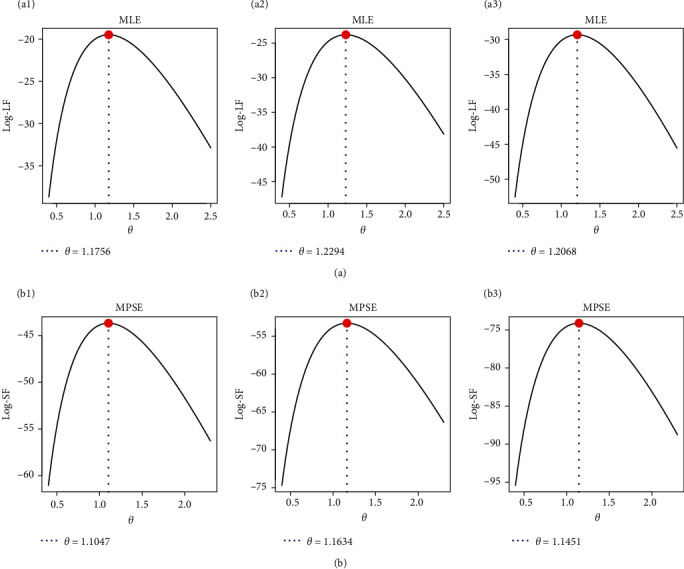
The log-LF (a: a1—S1, a2—S2, a3—S3) and log-SF (b: b1—S1, b2—S2, b3—S3) of *θ*, *R*(*t*), and *h*(*t*) from RME data.

**Figure 6:**
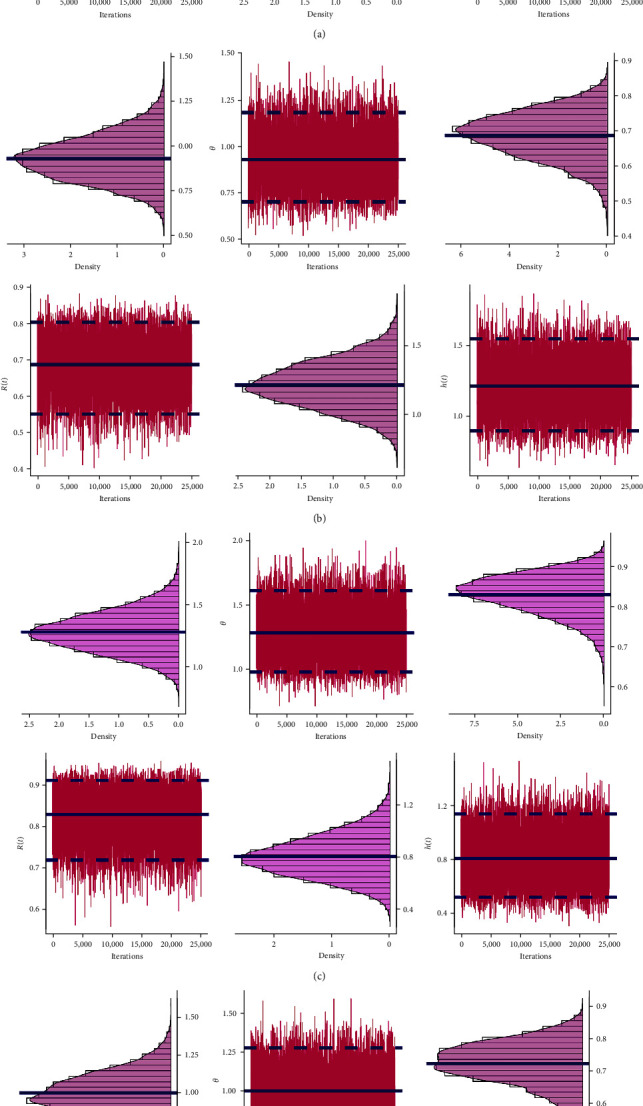
Two MCMC plots of *θ*, *R*(*t*), and *h*(*t*) based on S1 from RME data: (a) Bayes-LF; (b) Bayes-SF; (c) E-Bayes-LF; (d) E-Bayes-SF.

In order to investigate the existence and uniqueness characteristics of the proposed frequentist estimates of *θ*, the log-LF and log-SF curves of *θ* are shown in Figure 5. Based on all the samples listed in Table 13, it shows that the MLE or MPSE of *θ* might exist and are unique.  Figure 6 shows the density and trace plots of *θ*, *R*(*t*), and *h*(*t*) to illustrate the convergence of the MCMC. The dashed and solid lines, respectively, show the interval and point estimates for differentiation. Based on the remaining 25,000 MCMC iterations of each parameter, Figure 6 shows that the MCMC technique based on LF, SF, Bayes-LF, Bayes-SF, E-Bayes-LF, and E-Bayes-SF converges effectively using the data set of S1 (as an example) and that the burn-in phase is successful in mitigating the consequences of the specified initial values. All the MCMC iterations of *θ* are reasonably symmetrical, as seen in Figure 6, whereas those of *R*(*t*) and *h*(*t*) are negatively and positively skewed, respectively.

### 8.2. Physics Data Analysis

**Figure 7:**
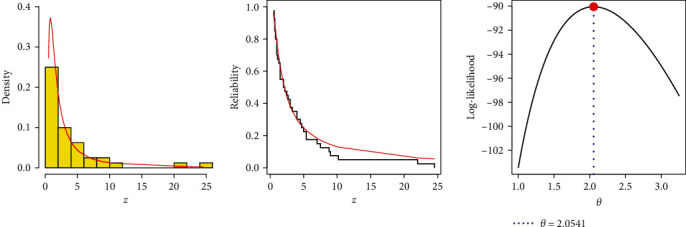
Graphics for goodness-of-fit of IL model using ART-ACT data.

The airborne communication transceiver is an ultra-high frequency transceiver that is intended for use in air traffic control communication as well as intercom communication between aircraft. We will be using a data set in this application that was reported by Jorgensen [48] and reanalyzed by Alotaibi et al. [25].  Table 15 shows 40 observations of the active repair times for an aerial communication transceiver (ART-ACT). Based on the entire ART-ACT data, the MLE (Std.Er) of *θ* and K–S (*p*-value) are 2.0542 (0.2609) and 0.0855 (0.9313), respectively. According to this finding, the ART-ACT data is fairly well-fitted by the IL distribution. The fitting plots in Figure 7 support our conclusion.

Again, to explain the usefulness of the proposed IL model based on ART-ACT data, we recompare the IL with the same six distributions mentioned in Subsection 8.1; see Table 16. It supports the same fact reached in Table 12 and demonstrates that the IL model is the better choice than others from ART-ACT data.

**Figure 8:**
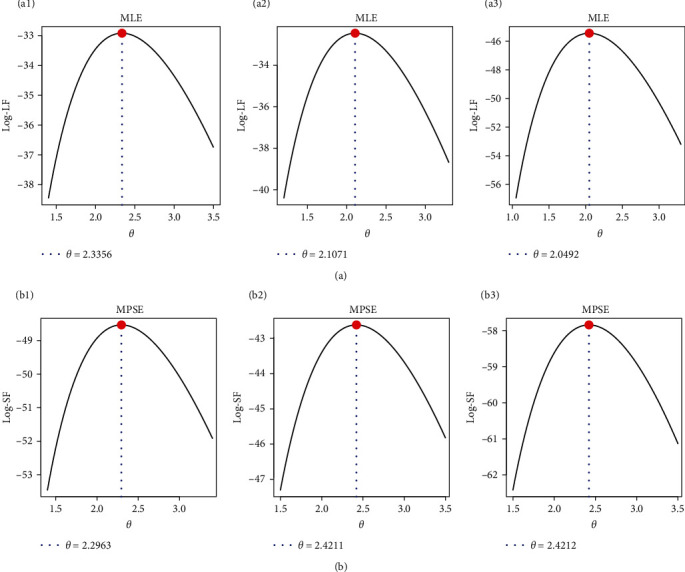
The log-LF (a: a1—S1, a2—S2, a3—S3) and log-SF (b: b1—S1, b2—S2, b3—S3) of *θ*, *R*(*t*), and *h*(*t*) from ART-ACT data.

**Figure 9:**
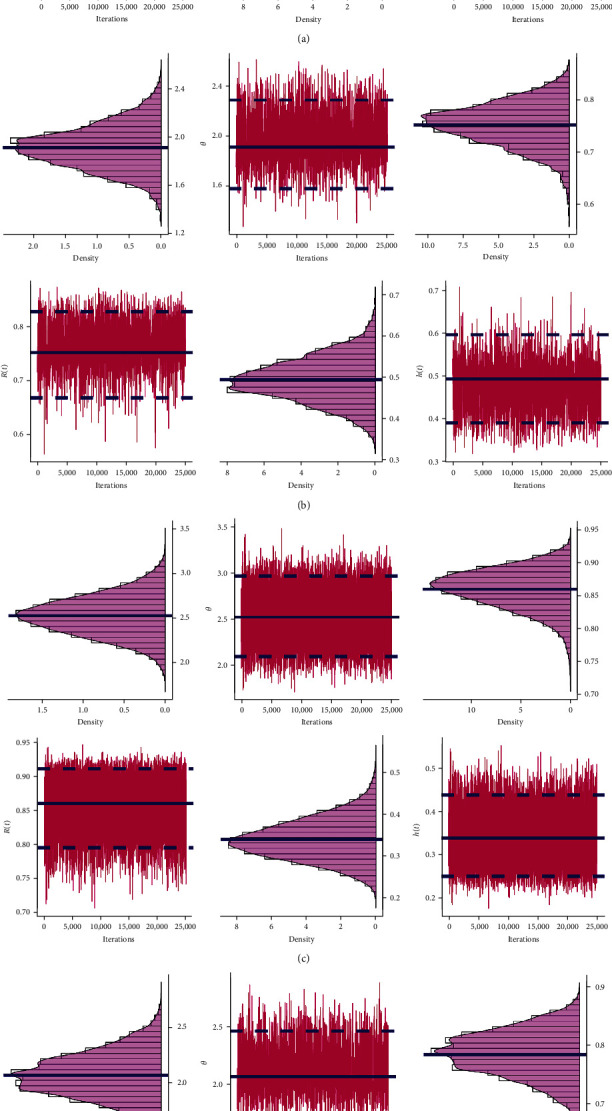
Two MCMC plots of *θ*, *R*(*t*), and *h*(*t*) based on S1 from ART-ACT data: (a) Bayes-LF; (b) Bayes-SF; (c) E-Bayes-LF; (d) E-Bayes-SF.

From the full ART-ACT data, with *m*=20, based on different options of *τ* and **S**, three artificial AT-IPC are acquired; see Table 17. Following the same MCMC settings reported in Section 8.1, when *c*=1 and *ɛ*_*i*_=0.75 for *i*=1, 2, both Bayes and E-Bayes estimates developed by LF and SF methods (developed in Sections 4 and 5) are obtained. From S*i* for *i*=1, 2, 3, in Table 17, all point and interval estimates of *θ*, *R*(*t*), and *h*(*t*) (at *t*=1) developed by frequentist and Bayes are computed; see Table 18. Results in Table 14 showed that the point estimates (including MLE, MPSE, Bayes-LF, Bayes-SF, E-Bayes-LF, and E-Bayes-SF) as well as the interval estimates (including ACI-LF, ACI-SF, BCI-LF, BCI-SF, E-BCI-LF, and E-BCI-SF) of *θ*, *R*(*t*), and *h*(*t*) are quite close to each other. The log-LF and log-SF curves of *θ* are shown in Figure 8 to verify the existence and uniqueness of the MLE and MPSE of *θ*. It shows that the MLE or MPSE of *θ* might exist and are unique based on the samples presented in Table 13. Based on S1 (for instance) from ART-ACT, Figure 9 displays the same facts as shown in Figure 6.

In conclusion, we can infer from the results of the engineering and physics evaluations that all of the inferential procedures suggested here work effectively with real-world data and offer a sufficient interpretation of the IL lifetime model when the necessary sample is obtained using the suggested censoring strategy.

## 9. Conclusions

In this research, based on adaptive Type-I progressive censoring, we proposed many point and interval estimators for the scale parameter and two reliability indicators where the basic distribution is the IL distribution. Two conventional and four Bayesian approaches are considered to accomplish this task, namely, ML, MPS, Bayesian using LF, Bayesian using SF, E-Bayesian using LF, and E-Bayesian using SF estimation methods. Both of the classical approaches are employed to look at the ACIs and the Bayesian and E-Bayesian perspectives are applied to get the Bayes and E-BCIs. To address the difficulties of theoretically comparing the various estimates, numerous simulations are run utilizing various performance standards and testing instances to compare the acquired point and interval estimates. The significance and feasibility of the examined approaches are demonstrated using two actual data sets from the domains of physics and engineering. According to the numerical results, the E-Bayesian estimations (point and interval) that use the SF as an observed data source outperform the classical estimations, Bayesian estimations using both classical functions, and E-Bayesian estimations using the LF.

## Figures and Tables

**Algorithm 1:**
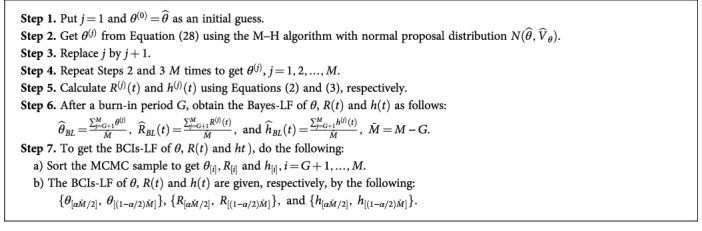
Steps to Generate and Compute Bayes Estimates.

**Algorithm 2:**
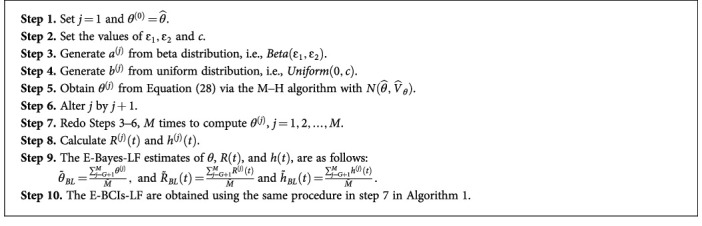
Steps to Generate and Compute E-bayes Estimates.

**Table 1:** Different simulation designs.

Test	*n*	*k*	**S**
(1)	40	20	(5^4^, 0^16^)
(2)	—	—	(0^8^, 5^4^, 0^8^)
(3)	—	—	(0^16^, 5^4^)
(4)	—	30	(5^2^, 0^28^)
(5)	—	—	(0^14^, 5^2^, 0^14^)
(6)	—	—	(0^28^, 5^2^)

(1)	80	40	(10^4^, 0^36^)
(2)	—	—	(0^18^, 5^4^, 0^18^)
(3)	—	—	(0^36^, 5^4^)
(4)	—	60	(10^2^, 0^58^)
(5)	—	—	(0^29^, 5^2^, 0^29^)
(6)	—	—	(0^58^, 5^2^)

**Table 2:** The Av.Es (1st Col.), RMSEs (2nd Col.), and MABs (3rd Col.) of *θ* when *τ*=0.5.

*n*	Test	MLE MPSE			Bayes-LF Bayes-SF			E-Bayes-LF E-Bayes-SF		
40	(1)	0.5042	0.0683	0.0532	0.5171	0.0570	0.0498	0.5213	0.0523	0.0456
		0.4858	0.0671	0.0574	0.5056	0.0635	0.0540	0.4993	0.0584	0.0523
	(2)	0.5036	0.0667	0.0518	0.5226	0.0553	0.0473	0.5256	0.0494	0.0396
		0.4885	0.0646	0.0512	0.5116	0.0569	0.0432	0.5060	0.0486	0.0402
	(3)	0.5080	0.0626	0.0487	0.5036	0.0533	0.0432	0.5075	0.0484	0.0391
		0.4936	0.0626	0.0488	0.5073	0.0530	0.0418	0.4976	0.0471	0.0389
	(4)	0.5077	0.0610	0.0475	0.4789	0.0485	0.0432	0.4844	0.0441	0.0352
		0.4912	0.0596	0.0477	0.4656	0.0481	0.0411	0.4705	0.0445	0.0354
	(5)	0.5066	0.0582	0.0447	0.4774	0.0452	0.0367	0.4838	0.0420	0.0343
		0.4923	0.0585	0.0466	0.4668	0.0450	0.0402	0.4708	0.0421	0.0342
	(6)	0.5080	0.0599	0.0467	0.4993	0.0437	0.0364	0.4977	0.0410	0.0325
		0.4939	0.0548	0.0464	0.4897	0.0414	0.0376	0.4891	0.0396	0.0321

80	(1)	0.5030	0.0520	0.0440	0.5091	0.0420	0.0327	0.5056	0.0404	0.0310
		0.4908	0.0501	0.0402	0.4937	0.0392	0.0371	0.4967	0.0372	0.0315
	(2)	0.5026	0.0497	0.0414	0.5050	0.0401	0.0319	0.5024	0.0384	0.0296
		0.4963	0.0484	0.0372	0.4917	0.0366	0.0349	0.4948	0.0353	0.0308
	(3)	0.5059	0.0465	0.0376	0.4562	0.0372	0.0309	0.4553	0.0355	0.0272
		0.5011	0.0450	0.0350	0.4439	0.0349	0.0332	0.4487	0.0334	0.0304
	(4)	0.5039	0.0453	0.0364	0.5157	0.0363	0.0291	0.5191	0.0342	0.0255
		0.4938	0.0433	0.0347	0.5072	0.0328	0.0316	0.5059	0.0323	0.0286
	(5)	0.5035	0.0429	0.0338	0.5174	0.0335	0.0283	0.5205	0.0315	0.0253
		0.4950	0.0423	0.0336	0.5098	0.0308	0.0314	0.5091	0.0285	0.0280
	(6)	0.5034	0.0412	0.0330	0.4773	0.0322	0.0265	0.4785	0.0292	0.0230
		0.4951	0.0397	0.0319	0.4703	0.0291	0.0286	0.4668	0.0263	0.0280

**Table 3:** The Av.Es (1st Col.), RMSEs (2nd Col.), and MABs (3rd Col.) of *θ* when *τ*=1.5.

*n*	Test	MLE MPSE			Bayes-LF Bayes-SF			E-Bayes-LF E-Bayes-SF		
40	(1)	0.5037	0.0674	0.0528	0.5210	0.0559	0.0451	0.5265	0.0501	0.0405
		0.4855	0.0664	0.0553	0.5096	0.0590	0.0526	0.5042	0.0538	0.0472
	(2)	0.5035	0.0661	0.0514	0.5229	0.0535	0.0434	0.5263	0.0494	0.0392
		0.4884	0.0641	0.0510	0.5121	0.0525	0.0428	0.5064	0.0484	0.0401
	(3)	0.5159	0.0616	0.0479	0.5083	0.0499	0.0424	0.5114	0.0473	0.0381
		0.5011	0.0616	0.0479	0.5110	0.0509	0.0416	0.5011	0.0470	0.0388
	(4)	0.5071	0.0604	0.0472	0.4790	0.0475	0.0397	0.4841	0.0427	0.0344
		0.4909	0.0595	0.0473	0.4661	0.0462	0.0368	0.4707	0.0418	0.0343
	(5)	0.5061	0.0560	0.0447	0.4778	0.0451	0.0366	0.4838	0.0413	0.0331
		0.4920	0.0559	0.0447	0.4667	0.0399	0.0345	0.4710	0.0384	0.0327
	(6)	0.5129	0.0560	0.0427	0.5012	0.0420	0.0351	0.5004	0.0409	0.0323
		0.4987	0.0517	0.0425	0.4922	0.0395	0.0342	0.4917	0.0363	0.0314

80	(1)	0.5029	0.0506	0.0401	0.5107	0.0398	0.0323	0.5063	0.0395	0.0310
		0.4909	0.0498	0.0400	0.4951	0.0369	0.0321	0.4979	0.0349	0.0301
	(2)	0.5025	0.0475	0.0374	0.5067	0.0393	0.0305	0.5046	0.0383	0.0296
		0.4962	0.0468	0.0369	0.4940	0.0339	0.0313	0.4966	0.0318	0.0288
	(3)	0.5176	0.0453	0.0345	0.4623	0.0369	0.0303	0.4610	0.0352	0.0269
		0.5126	0.0436	0.0348	0.4491	0.0319	0.0302	0.4545	0.0293	0.0277
	(4)	0.5041	0.0430	0.0338	0.5211	0.0347	0.0288	0.5232	0.0328	0.0253
		0.4941	0.0424	0.0336	0.5115	0.0284	0.0290	0.5097	0.0258	0.0265
	(5)	0.5036	0.0419	0.0330	0.5199	0.0334	0.0270	0.5229	0.0306	0.0232
		0.4951	0.0402	0.0331	0.5119	0.0268	0.0281	0.5120	0.0250	0.0245
	(6)	0.5084	0.0401	0.0313	0.4809	0.0318	0.0260	0.4834	0.0279	0.0218
		0.4997	0.0389	0.0313	0.4740	0.0236	0.0272	0.4708	0.0213	0.0230

**Table 4:** The Av.Es (1st Col.), RMSEs (2nd Col.), and MABs (3rd Col.) of *R*(*t*) when *τ*=0.5.

*n*	Test	MLE MPSE			Bayes-LF Bayes-SF			E-Bayes-LF E-Bayes-SF		
40	(1)	0.9615	0.2409	0.2078	0.9706	0.2276	0.1849	0.9688	0.2164	0.1668
		0.9669	0.1989	0.1632	0.9729	0.1820	0.1412	0.9736	0.1690	0.1262
	(2)	0.9635	0.2098	0.1523	0.9722	0.1952	0.1482	0.9707	0.1757	0.1343
		0.9678	0.1803	0.1345	0.9743	0.1518	0.1255	0.9747	0.1437	0.1140
	(3)	0.9651	0.1917	0.1478	0.9711	0.1878	0.1374	0.9683	0.1684	0.1291
		0.9691	0.1695	0.1245	0.9702	0.1410	0.1171	0.9711	0.1322	0.1038
	(4)	0.9635	0.1891	0.1392	0.9579	0.1589	0.1229	0.9598	0.1417	0.1115
		0.9681	0.1642	0.1243	0.9626	0.1342	0.1012	0.9645	0.1246	0.0994
	(5)	0.9645	0.1812	0.1345	0.9585	0.1328	0.1019	0.9601	0.1182	0.0912
		0.9685	0.1583	0.1209	0.9622	0.1206	0.0994	0.9645	0.1113	0.0929
	(6)	0.9653	0.1767	0.1329	0.9663	0.1175	0.0929	0.9657	0.1141	0.0882
		0.9691	0.1549	0.1160	0.9689	0.1147	0.0961	0.9686	0.1078	0.0883

80	(1)	0.9653	0.1563	0.1174	0.9673	0.1127	0.0856	0.9683	0.1110	0.0835
		0.9688	0.1372	0.1057	0.9718	0.1083	0.0871	0.9710	0.1036	0.0813
	(2)	0.9676	0.1405	0.1055	0.9670	0.1070	0.0836	0.9680	0.1034	0.0810
		0.9694	0.1266	0.0974	0.9710	0.0988	0.0798	0.9703	0.0972	0.0778
	(3)	0.9692	0.1325	0.0961	0.9503	0.1024	0.0794	0.9525	0.1003	0.0765
		0.9705	0.1215	0.0902	0.9555	0.0953	0.0758	0.9550	0.0913	0.0747
	(4)	0.9666	0.1244	0.0941	0.9709	0.1012	0.0771	0.9710	0.0961	0.0727
		0.9694	0.1139	0.0880	0.9732	0.0901	0.0739	0.9742	0.0885	0.0722
	(5)	0.9674	0.1207	0.0919	0.9718	0.0952	0.0753	0.9719	0.0919	0.0703
		0.9697	0.1109	0.0866	0.9738	0.0883	0.0706	0.9746	0.0827	0.0697
	(6)	0.9675	0.1104	0.0872	0.9607	0.0914	0.0725	0.9594	0.0827	0.0627
		0.9698	0.1047	0.0831	0.9628	0.0845	0.0668	0.9632	0.0783	0.0654

**Table 5:** The Av.Es (1st Col.), RMSEs (2nd Col.), and MABs (3rd Col.) of *R*(*t*) when *τ*=1.5.

*n*	Test	MLE MPSE			Bayes-LF Bayes-SF			E-Bayes-LF E-Bayes-SF		
40	(1)	0.9615	0.2269	0.1866	0.9716	0.2197	0.1657	0.9702	0.1954	0.1564
		0.9669	0.1895	0.1451	0.9738	0.1811	0.1357	0.9747	0.1676	0.1242
	(2)	0.9635	0.2090	0.1520	0.9724	0.1922	0.1462	0.9708	0.1748	0.1337
		0.9678	0.1797	0.1340	0.9744	0.1511	0.1152	0.9749	0.1435	0.1012
	(3)	0.9672	0.1916	0.1430	0.9721	0.1848	0.1342	0.9693	0.1682	0.1288
		0.9711	0.1644	0.1247	0.9714	0.1351	0.1144	0.9721	0.1272	0.0994
	(4)	0.9634	0.1882	0.1383	0.9582	0.1456	0.1117	0.9599	0.1322	0.1021
		0.9681	0.1641	0.1237	0.9626	0.1263	0.0993	0.9645	0.1205	0.0939
	(5)	0.9645	0.1690	0.1267	0.9584	0.1292	0.0991	0.9602	0.1153	0.0895
		0.9684	0.1507	0.1169	0.9623	0.1186	0.0960	0.9645	0.1106	0.0901
	(6)	0.9668	0.1624	0.1241	0.9670	0.1148	0.0858	0.9665	0.1094	0.0864
		0.9706	0.1456	0.1140	0.9695	0.1144	0.0920	0.9694	0.1070	0.0878

80	(1)	0.9654	0.1555	0.1168	0.9678	0.1107	0.0851	0.9686	0.1071	0.0821
		0.9688	0.1366	0.1056	0.9722	0.1047	0.0832	0.9711	0.1023	0.0804
	(2)	0.9676	0.1395	0.1046	0.9677	0.1060	0.0828	0.9685	0.1011	0.0803
		0.9694	0.1260	0.0972	0.9714	0.0987	0.0798	0.9709	0.0958	0.0774
	(3)	0.9721	0.1245	0.0959	0.9525	0.1007	0.0789	0.9548	0.0978	0.0758
		0.9733	0.1184	0.0900	0.9579	0.0945	0.0757	0.9571	0.0895	0.0746
	(4)	0.9667	0.1171	0.0941	0.9720	0.0988	0.0759	0.9720	0.0948	0.0717
		0.9694	0.1121	0.0863	0.9744	0.0890	0.0738	0.9751	0.0833	0.0714
	(5)	0.9674	0.1113	0.0860	0.9723	0.0894	0.0726	0.9727	0.0851	0.0671
		0.9697	0.1082	0.0830	0.9744	0.0867	0.0703	0.9751	0.0815	0.0669
	(6)	0.9690	0.1038	0.0836	0.9620	0.0862	0.0676	0.9608	0.0826	0.0614
		0.9712	0.1017	0.0828	0.9639	0.0843	0.0656	0.9647	0.0783	0.0630

**Table 6:** The Av.Es (1st Col.), RMSEs (2nd Col.), and MABs (3rd Col.) of *h*(*t*) when *τ*=0.5.

*n*	Test	MLE MPSE			Bayes-LF Bayes-SF			E-Bayes-LF E-Bayes-SF		
40	(1)	1.5239	0.7197	0.6311	1.2631	0.6762	0.5663	1.3206	0.6518	0.5218
		1.3544	0.6001	0.4650	1.1802	0.5911	0.4489	1.1552	0.5756	0.4086
	(2)	1.4728	0.6306	0.4819	1.2109	0.5843	0.4564	1.2592	0.5317	0.4176
		1.3349	0.5666	0.4418	1.1331	0.4866	0.3865	1.1167	0.4225	0.3471
	(3)	1.4226	0.5983	0.4654	1.2462	0.5639	0.4439	1.3369	0.5116	0.4024
		1.2951	0.5243	0.4200	1.2769	0.4651	0.3671	1.2468	0.4135	0.3351
	(4)	1.4649	0.5767	0.4445	1.6608	0.4835	0.3833	1.6047	0.4378	0.3509
		1.3172	0.5201	0.4093	1.5181	0.4142	0.3423	1.4599	0.4105	0.3202
	(5)	1.4391	0.5568	0.4315	1.6448	0.4162	0.3284	1.5972	0.3735	0.2956
		1.3112	0.5071	0.4009	1.5322	0.4072	0.3285	1.4626	0.3816	0.3170
	(6)	1.4179	0.5470	0.4274	1.4052	0.3719	0.2917	1.4192	0.3669	0.2873
		1.2932	0.5001	0.3989	1.3186	0.3994	0.3173	1.3291	0.3734	0.3068

80	(1)	1.4239	0.4855	0.3776	1.3704	0.3611	0.2820	1.3403	0.3511	0.2794
		1.3136	0.4417	0.3497	1.2251	0.3487	0.2777	1.2534	0.3373	0.2727
	(2)	1.3573	0.4416	0.3424	1.3828	0.3446	0.2751	1.3528	0.3432	0.2714
		1.2991	0.4103	0.3239	1.2554	0.3325	0.2695	1.2787	0.3276	0.2665
	(3)	1.3084	0.3997	0.3150	1.8934	0.3337	0.2660	1.8310	0.3326	0.2596
		1.2658	0.3762	0.3005	1.7428	0.3218	0.2651	1.7573	0.3199	0.2570
	(4)	1.3877	0.3955	0.3094	1.2515	0.3287	0.2630	1.2530	0.3232	0.2560
		1.2970	0.3720	0.2934	1.1764	0.3115	0.2590	1.1453	0.3091	0.2565
	(5)	1.3658	0.3831	0.3002	1.2252	0.3222	0.2601	1.2228	0.2979	0.2405
		1.2900	0.3614	0.2884	1.1582	0.2975	0.2407	1.1317	0.2922	0.2345
	(6)	1.3619	0.3615	0.2901	1.5855	0.3193	0.2557	1.6258	0.2826	0.2319
		1.2879	0.3487	0.2798	1.5192	0.2831	0.2231	1.5073	0.2771	0.2122

**Table 7:** The Av.Es (1st Col.), RMSEs (2nd Col.), and MABs (3rd Col.) of *h*(*t*) when *τ*=1.5.

*n*	Test	MLE MPSE			Bayes-LF Bayes-SF			E-Bayes-LF E-Bayes-SF		
40	(1)	1.5247	0.6735	0.5705	1.2286	0.6604	0.5181	1.2770	0.5926	0.5042
		1.3563	0.5887	0.4597	1.1488	0.5489	0.4240	1.1144	0.5124	0.3618
	(2)	1.4732	0.6283	0.4809	1.2054	0.5763	0.4512	1.2552	0.5292	0.4161
		1.3355	0.5644	0.4400	1.1302	0.4677	0.3649	1.1108	0.4189	0.3429
	(3)	1.3546	0.5830	0.4542	1.2152	0.5465	0.4401	1.3056	0.5110	0.4015
		1.2290	0.5216	0.4099	1.2373	0.4647	0.3623	1.2134	0.4121	0.3235
	(4)	1.4666	0.5737	0.4413	1.6541	0.4460	0.3506	1.6021	0.4095	0.3225
		1.3208	0.5120	0.4077	1.5180	0.4115	0.3230	1.4617	0.3911	0.3123
	(5)	1.4406	0.5289	0.4133	1.6463	0.4071	0.3210	1.5956	0.3670	0.2918
		1.3138	0.4944	0.3948	1.5284	0.3991	0.3167	1.4622	0.3790	0.3058
	(6)	1.3707	0.5159	0.4039	1.3832	0.3650	0.2846	1.3951	0.3557	0.2827
		1.2482	0.4790	0.3839	1.3014	0.3765	0.2988	1.3057	0.3572	0.2947

80	(1)	1.4225	0.4832	0.3755	1.3567	0.3495	0.2796	1.3289	0.3411	0.2762
		1.3135	0.4400	0.3492	1.2113	0.3410	0.2754	1.2484	0.3324	0.2677
	(2)	1.3577	0.4387	0.3396	1.3610	0.3426	0.2743	1.3351	0.3341	0.2648
		1.2996	0.4085	0.3231	1.2414	0.3292	0.2660	1.2587	0.3238	0.2628
	(3)	1.2118	0.3980	0.3145	1.8296	0.3323	0.2651	1.7631	0.3288	0.2564
		1.1701	0.3744	0.2996	1.6729	0.3198	0.2590	1.6930	0.3119	0.2551
	(4)	1.3842	0.3896	0.3073	1.2154	0.3216	0.2603	1.2197	0.3193	0.2544
		1.2948	0.3702	0.2904	1.1337	0.3079	0.2515	1.1129	0.3041	0.2453
	(5)	1.3649	0.3592	0.2860	1.2074	0.3187	0.2569	1.1985	0.2925	0.2393
		1.2897	0.3522	0.2869	1.1379	0.2947	0.2330	1.1124	0.2895	0.2239
	(6)	1.3155	0.3453	0.2814	1.5473	0.3183	0.2516	1.5827	0.2806	0.2286
		1.2429	0.3462	0.2795	1.4832	0.2786	0.2217	1.4591	0.2758	0.2081

**Table 8:** The ACLs for 95% interval estimates of *θ*.

*n*	Test	ACI-LF	BCI-LF	E-BCI-LF	ACI-SF	BCI-SF	E-BCI-SF
*τ*=0.5							

40	(1)	0.270	0.185	0.179	0.257	0.179	0.165
	(2)	0.254	0.174	0.168	0.244	0.169	0.158
	(3)	0.244	0.169	0.154	0.232	0.153	0.151
	(4)	0.241	0.165	0.149	0.227	0.150	0.148
	(5)	0.232	0.160	0.142	0.222	0.149	0.146
	(6)	0.221	0.155	0.139	0.214	0.148	0.140

80	(1)	0.199	0.146	0.138	0.193	0.146	0.139
	(2)	0.181	0.139	0.135	0.176	0.145	0.136
	(3)	0.172	0.135	0.132	0.167	0.142	0.131
	(4)	0.168	0.129	0.127	0.163	0.141	0.128
	(5)	0.163	0.125	0.120	0.161	0.137	0.115
	(6)	0.160	0.120	0.113	0.159	0.131	0.111

*τ*=1.5							

40	(1)	0.267	0.184	0.174	0.256	0.166	0.159
	(2)	0.251	0.171	0.162	0.242	0.167	0.153
	(3)	0.241	0.165	0.154	0.230	0.152	0.150
	(4)	0.238	0.164	0.145	0.225	0.150	0.147
	(5)	0.223	0.150	0.140	0.192	0.146	0.138
	(6)	0.213	0.146	0.138	0.175	0.141	0.136

80	(1)	0.198	0.145	0.137	0.167	0.139	0.131
	(2)	0.179	0.138	0.133	0.158	0.132	0.125
	(3)	0.170	0.133	0.131	0.150	0.130	0.120
	(4)	0.167	0.128	0.125	0.144	0.128	0.117
	(5)	0.161	0.123	0.119	0.136	0.118	0.114
	(6)	0.156	0.119	0.111	0.130	0.113	0.108

**Table 9:** The ACLs for 95% interval estimates of *R*(*t*).

*n*	Test	ACI-LF	BCI-LF	E-BCI-LF	ACI-SF	BCI-SF	E-BCI-SF
*τ*=0.5							

40	(1)	0.081	0.057	0.052	0.074	0.052	0.049
	(2)	0.074	0.055	0.050	0.067	0.051	0.047
	(3)	0.071	0.050	0.048	0.065	0.048	0.045
	(4)	0.069	0.045	0.044	0.062	0.045	0.044
	(5)	0.067	0.043	0.042	0.061	0.043	0.040
	(6)	0.065	0.042	0.041	0.060	0.040	0.038

80	(1)	0.057	0.041	0.039	0.053	0.039	0.037
	(2)	0.054	0.040	0.038	0.048	0.038	0.035
	(3)	0.051	0.039	0.038	0.045	0.036	0.034
	(4)	0.047	0.037	0.036	0.043	0.033	0.032
	(5)	0.045	0.036	0.032	0.042	0.033	0.029
	(6)	0.044	0.034	0.031	0.040	0.031	0.027

*τ*=1.5							

40	(1)	0.078	0.057	0.051	0.071	0.052	0.048
	(2)	0.073	0.055	0.049	0.067	0.049	0.046
	(3)	0.071	0.048	0.046	0.061	0.047	0.045
	(4)	0.068	0.044	0.041	0.058	0.045	0.043
	(5)	0.065	0.042	0.038	0.056	0.042	0.040
	(6)	0.063	0.041	0.035	0.055	0.040	0.038

80	(1)	0.057	0.040	0.031	0.053	0.037	0.034
	(2)	0.050	0.039	0.029	0.047	0.035	0.031
	(3)	0.047	0.038	0.027	0.045	0.034	0.028
	(4)	0.045	0.037	0.025	0.043	0.033	0.026
	(5)	0.043	0.035	0.023	0.041	0.032	0.024
	(6)	0.040	0.033	0.022	0.039	0.030	0.023

**Table 10:** The ACLs for 95% interval estimates of *h*(*t*).

*n*	Test	ACI-LF	BCI-LF	E-BCI-LF	ACI-SF	BCI-SF	E-BCI-SF
*τ*=0.5							

40	(1)	2.497	1.707	1.579	2.355	1.615	1.508
	(2)	2.291	1.667	1.496	2.162	1.599	1.479
	(3)	2.214	1.466	1.449	2.100	1.453	1.397
	(4)	2.110	1.426	1.408	2.002	1.387	1.325
	(5)	2.100	1.410	1.374	1.991	1.353	1.309
	(6)	2.070	1.331	1.305	1.913	1.306	1.297

80	(1)	1.810	1.327	1.286	1.739	1.273	1.267
	(2)	1.625	1.300	1.268	1.566	1.257	1.248
	(3)	1.519	1.243	1.225	1.484	1.218	1.153
	(4)	1.471	1.238	1.214	1.425	1.123	1.107
	(5)	1.452	1.224	1.157	1.418	1.115	1.096
	(6)	1.434	1.174	1.070	1.406	1.077	1.046

*τ*=1.5							

40	(1)	2.487	1.694	1.572	2.347	1.613	1.453
	(2)	2.277	1.647	1.491	2.152	1.592	1.430
	(3)	2.102	1.451	1.423	1.996	1.400	1.377
	(4)	2.036	1.396	1.377	1.926	1.385	1.300
	(5)	2.036	1.353	1.337	1.926	1.295	1.284
	(6)	2.009	1.318	1.297	1.896	1.268	1.254

80	(1)	1.799	1.290	1.270	1.730	1.255	1.225
	(2)	1.611	1.275	1.258	1.553	1.248	1.209
	(3)	1.515	1.224	1.220	1.480	1.175	1.148
	(4)	1.463	1.208	1.193	1.418	1.108	1.093
	(5)	1.414	1.184	1.096	1.369	1.084	1.082
	(6)	1.367	1.171	1.059	1.339	1.073	1.030

**Table 11:** Thirty failure times of repairable mechanical equipment.

0.11	0.30	0.40	0.45	0.59	0.63	0.70	0.71	0.74	0.77
0.94	1.06	1.17	1.23	1.23	1.24	1.43	1.46	1.49	1.74
1.82	1.86	1.97	2.23	2.37	2.46	2.63	3.46	4.36	4.73

**Table 12:** Fitting outputs of the IL and its competitors from RME data.

Model	$\hat{\gamma }$		$\hat{\theta }$		*A*	*B*	*CA*	*HQ*	K–S(*p*-Value)
	Est.	Std.Er	Est.	Std.Er					
IL	—	—	1.160	0.162	95.866	97.267	96.009	96.314	0.141 (0.588)
IW	1.073	0.131	0.752	0.157	96.751	99.554	97.196	97.648	0.144 (0.585)
ILomax	7.667	8.887	0.120	0.155	96.027	98.829	96.471	96.923	0.190 (0.231)
IChen	0.446	0.092	0.488	0.045	109.42	112.22	109.86	110.31	0.234 (0.075)
IER	0.364	0.078	0.102	0.035	109.77	112.57	110.21	110.67	0.260 (0.035)
INH	0.852	0.235	1.035	0.513	96.740	99.543	97.185	97.637	0.179 (0.294)
APIE	0.751	1.757	0.858	0.559	97.053	99.856	97.498	97.950	0.150 (0.506)

**Table 13:** Three AT-IPC samples from RME data.

Sample	Scheme	*τ*(*k*)	*S* ^*∗*^	Data
S1	(3^5^, 0^10^)	1.5 (10)	5	0.11, 0.30, 0.45, 0.59, 0.74, 0.77, 1.06, 1.17, 1.23, 1.46
S2	(0^5^, 3^5^, 0^5^)	1.8 (12)	3	0.11, 0.30, 0.40, 0.45, 0.59, 0.63, 0.71, 0.74, 0.94, 1.17, 1.49, 1.74
S3	(0^10^, 3^5^)	2.1 (17)	1	0.11, 0.30, 0.40, 0.45, 0.59, 0.63, 0.70, 0.71, 0.74, 0.77, 0.94, 1.06, 1.24, 1.46, 1.74, 1.86, 1.97

**Table 14:** Estimates of *θ*, *R*(*t*), and *h*(*t*) from RME data.

Sample	Par.	MLE Bayes-LF E-Bayes-LF		MPSE Bayes-SF E-Bayes-SF		95% ACI-LF 95% BCI-LF 95% E-BCI-LF			95% ACI-SF 95% BCI-SF 95% E-BCI-SF		
		Est.	Std.Er	Est.	Std.Er	Low.	Upp.	Width	Low.	Upp.	Width
S1	*θ*	1.1756	0.1855	1.1047	0.1739	0.8120	1.5391	0.7271	0.7639	1.4455	0.6817
		1.1908	0.1502	0.9293	0.2146	0.9110	1.4983	0.5873	0.7027	1.1825	0.4798
		1.2830	0.1857	1.0015	0.1521	0.9807	1.6121	0.6314	0.7542	1.2773	0.5231
	*R*(0.5)	0.8018	0.0661	0.7750	0.0696	0.6723	0.9313	0.2590	0.6385	0.9115	0.2730
		0.8007	0.0527	0.6879	0.1089	0.6841	0.8901	0.2060	0.5523	0.8043	0.2519
		0.8302	0.0573	0.7234	0.0729	0.7201	0.9111	0.1910	0.5885	0.8351	0.2466
	*h*(0.5)	0.9056	0.2020	0.9855	0.2024	0.5097	1.3016	0.7918	0.5887	1.3823	0.7936
		0.9006	0.1598	1.2155	0.2845	0.6050	1.2318	0.6268	0.8981	1.5453	0.6472
		0.8071	0.1835	1.1216	0.1946	0.5213	1.1382	0.6169	0.8001	1.4630	0.6629

S2	*θ*	1.2294	0.1778	1.1635	0.1683	0.8810	1.5778	0.6968	0.8335	1.4934	0.6599
		1.2433	0.1463	0.9894	0.2134	0.9703	1.5397	0.5694	0.7633	1.2483	0.4850
		1.3397	0.1842	1.0689	0.1540	1.0455	1.6590	0.6135	0.8202	1.3419	0.5217
	*R*(0.5)	0.8201	0.0578	0.7974	0.0612	0.7068	0.9335	0.2267	0.6775	0.9173	0.2398
		0.8189	0.0472	0.7186	0.0986	0.7149	0.8983	0.1833	0.5946	0.8262	0.2315
		0.8471	0.0518	0.7542	0.0667	0.7501	0.9186	0.1684	0.6313	0.8534	0.2221
	*h*(0.5)	0.8485	0.1838	0.9189	0.1855	0.4883	1.2086	0.7204	0.5554	1.2824	0.7270
		0.8448	0.1484	1.1356	0.2683	0.5735	1.1518	0.5784	0.8291	1.4488	0.6196
		0.7521	0.1724	1.0375	0.1859	0.4898	1.0562	0.5664	0.7385	1.3621	0.6237

S3	*θ*	1.2068	0.1709	1.1451	0.1624	0.8719	1.5418	0.6698	0.8268	1.4634	0.6366
		1.2198	0.1424	0.9811	0.2038	0.9538	1.5091	0.5553	0.7543	1.2293	0.4750
		1.3144	0.1796	1.0572	0.1509	1.0277	1.6260	0.5983	0.8127	1.3245	0.5118
	*R*(0.5)	0.8126	0.0578	0.7907	0.0608	0.6995	0.9258	0.2264	0.6714	0.9099	0.2385
		0.8114	0.0477	0.7148	0.0963	0.7066	0.8923	0.1857	0.5885	0.8201	0.2316
		0.8402	0.0526	0.7494	0.0666	0.7422	0.9134	0.1712	0.6267	0.8487	0.2220
	*h*(0.5)	0.8721	0.1806	0.9393	0.1821	0.5182	1.2260	0.7079	0.5825	1.2962	0.7137
		0.8686	0.1477	1.1460	0.2595	0.5966	1.1737	0.5771	0.8486	1.4629	0.6143
		0.7754	0.1725	1.0513	0.1842	0.5117	1.0783	0.5666	0.7546	1.3732	0.6186

**Table 15:** Forty records of ART-ACT.

0.50	0.60	0.60	0.70	0.70	0.70	0.80	0.80	1.00	1.00
1.00	1.00	1.10	1.30	1.50	1.50	1.50	1.50	2.00	2.00
2.20	2.50	2.70	3.00	3.00	3.30	4.00	4.00	4.50	4.70
5.00	5.40	5.40	7.00	7.50	8.80	9.00	10.2	22.0	24.50

**Table 16:** Fitting outputs of the IL and its competitors from ART-ACT data.

Model	$\hat{\gamma }$		$\hat{\theta }$		*A*	*B*	*CA*	*HQ*	K–S(*p*-value)
	Est.	Std.Er	Est.	Std.Er					
IL	0.000	0.000	2.054	0.261	182.108	183.796	182.213	182.718	0.086 (0.931)
IW	1.208	0.152	1.569	0.248	182.898	186.276	183.222	184.119	0.095 (0.861)
ILomax	28.36	61.69	0.056	0.125	185.463	188.840	185.787	186.684	0.097 (0.849)
IChen	0.842	0.134	0.868	0.111	183.338	186.716	183.662	184.559	0.098 (0.836)
IER	0.472	0.087	0.847	0.220	183.198	186.576	183.522	184.419	0.104 (0.777)
INH	2.773	3.107	0.391	0.536	182.870	186.248	183.194	184.091	0.094 (0.868)
APIE	0.348	0.323	1.965	0.446	183.839	187.217	184.163	185.060	0.089 (0.908)

**Table 17:** Three AT-IPC samples from ART-ACT data.

Sample	Scheme	*τ*(*k*)	*S* ^*∗*^	Data
S1	(5^4^, 0^16^)	4.8 (15)	5	0.50, 0.60, 0.70, 0.80, 1.00, 1.00, 1.30, 1.50, 1.50, 2.00,
				2.70, 3.00, 3.30, 4.00, 4.70

S2	(0^8^, 5^4^, 0^8^)	3.4 (17)	3	0.50, 0.60, 0.60, 0.70, 0.70, 0.70, 0.80, 0.80, 1.00, 1.00,
				1.30, 1.50, 1.50, 2.00, 2.50, 2.70, 3.30

S3	(0^16^, 5^4^)	4.6 (24)	1	0.50, 0.60, 0.60, 0.70, 0.70, 0.70, 0.80, 0.80, 1.00, 1.00,
				1.00, 1.00, 1.10, 1.30, 1.50, 1.50, 1.50, 2.00, 2.20, 2.70,
				3.00, 3.30, 4.00, 4.50

$S^{*}=n-k-\sum_{i=1}^{m-1}S_{i}$ .

**Table 18:** Estimates of *θ*, *R*(*t*), and *h*(*t*) from ART-ACT data.

Sample	Par.	MLE Bayes-LF E-Bayes-LF		MPSE Bayes-SF E-Bayes-SF		95% ACI-LF 95% BCI-LF 95% E-BCI-LF			95% ACI-SF 95% BCI-SF 95% E-BCI-SF		
		Est.	Std.Er	Est.	Std.Er	Low.	Upp.	Width	Low.	Upp.	Width
S1	*θ*	2.3357	0.3500	2.2963	0.3535	1.6497	3.0216	1.3719	1.6034	2.9893	1.3858
		2.3430	0.2049	1.9183	0.4197	1.9493	2.7566	0.8073	1.5790	2.2863	0.7073
		2.5245	0.2858	2.0669	0.2464	2.1004	2.9702	0.8699	1.7014	2.4635	0.7621
	*R*(1)	0.8355	0.0545	0.8293	0.0571	0.7287	0.9424	0.2137	0.7174	0.9412	0.2238
		0.8337	0.0323	0.7533	0.0864	0.7635	0.8899	0.1264	0.6676	0.8276	0.1601
		0.8596	0.0393	0.7847	0.0502	0.7947	0.9103	0.1157	0.7027	0.8543	0.1516
	*h*(1)	0.3787	0.0835	0.3882	0.0861	0.2151	0.5424	0.3273	0.2195	0.5569	0.3374
		0.3796	0.0488	0.4918	0.1165	0.2887	0.4804	0.1917	0.3906	0.5971	0.2065
		0.3386	0.0630	0.4500	0.0679	0.2504	0.4383	0.1879	0.3492	0.5564	0.2072

S2	*θ*	2.1070	0.2805	2.4210	0.3635	1.5573	2.6567	1.0995	1.7085	3.1335	1.4250
		2.1154	0.1872	2.0318	0.4305	1.7588	2.4938	0.7351	1.6917	2.4074	0.7157
		2.2793	0.2598	2.1893	0.2533	1.8951	2.6871	0.7920	1.8228	2.5940	0.7712
	*R*(1)	0.7959	0.0537	0.8483	0.0524	0.6907	0.9012	0.2105	0.7456	0.9510	0.2053
		0.7946	0.0360	0.7780	0.0798	0.7179	0.8585	0.1405	0.7000	0.8463	0.1463
		0.8235	0.0443	0.8080	0.0464	0.7513	0.8823	0.1310	0.7341	0.8713	0.1373
	*h*(1)	0.4366	0.0751	0.3588	0.0830	0.2894	0.5838	0.2943	0.1962	0.5215	0.3252
		0.4366	0.0499	0.4594	0.1127	0.3424	0.5380	0.1956	0.3619	0.5596	0.1976
		0.3949	0.0648	0.4175	0.0658	0.3021	0.4963	0.1942	0.3211	0.5181	0.1970

S3	*θ*	2.0492	0.2638	2.4212	0.3632	1.5322	2.5662	1.0339	1.7094	3.1330	1.4236
		2.0574	0.1820	2.0293	0.4334	1.7078	2.4237	0.7158	1.6918	2.4075	0.7157
		2.2169	0.2526	2.1866	0.2540	1.8402	2.6115	0.7713	1.8229	2.5940	0.7711
	*R*(1)	0.7846	0.0532	0.8483	0.0523	0.6804	0.8888	0.2084	0.7458	0.9509	0.2051
		0.7833	0.0368	0.7775	0.0804	0.7044	0.8487	0.1443	0.7000	0.8463	0.1463
		0.8130	0.0455	0.8075	0.0466	0.7383	0.8735	0.1351	0.7341	0.8714	0.1372
	*h*(1)	0.4523	0.0726	0.3588	0.0829	0.3099	0.5947	0.2847	0.1963	0.5212	0.3249
		0.4522	0.0499	0.4601	0.1135	0.3582	0.5543	0.1961	0.3619	0.5595	0.1976
		0.4104	0.0650	0.4182	0.0660	0.3175	0.5128	0.1954	0.3211	0.5181	0.1970

## Data Availability

The authors confirm that the data supporting the findings of this study are available within the article.
